# Polymer Matrix‐Based 3D Culture Significantly Enhances the Differentiation and Immunomodulatory Functions of Human Adipose‐Derived Stem Cells

**DOI:** 10.1002/advs.202518704

**Published:** 2026-03-31

**Authors:** Changjin Seo, Dohyeon Kim, Junhyuk Song, Sun‐Young Kim, Youngju Son, Afia Tasnim Rahman, Sangyong Jon

**Affiliations:** ^1^ Department of Biological Sciences KAIST Institute for the BioCentury Korea Advanced Institute of Science and Technology (KAIST) Daejeon Republic of Korea; ^2^ Center For Precision Bio‐Nanomedicine Korea Advanced Institute of Science and Technology (KAIST) Daejeon Republic of Korea; ^3^ InnoCORE AI‐CRED Institute Korea Advanced Institute of Science and Technology (KAIST) Daejeon Republic of Korea

**Keywords:** anti‐inflammation therapy, cell therapy, human adipose‐derived stem cell, polymer matrix, spheroid culture

## Abstract

Human adipose‐derived stem cells (hADSCs) possess immunomodulatory and multipotent properties, making them attractive candidates for regenerative therapies. However, conventional tissue culture plate (TCP)‐based 2D adherent cultures or widely used ultra‐low attachment (ULA) spheroid systems often compromise differentiation potential and therapeutic efficacy of hADSCs. Here, we report a synthetic polymer matrix, poly‐Z, that enables the formation of hADSC spheroids with significantly enhanced biological functions. Compared to ULA or TCP culture systems, poly‐Z‐cultured hADSC spheroids exhibited improved cell viability, enriched extracellular matrix deposition, elevated pluripotency marker expression, and superior tri‐lineage differentiation capacity. Notably, poly‐Z spheroids displayed a distinct immunomodulatory signature characterized by significantly elevated secretion of indoleamine‐pyrrole 2,3‐dioxygenase (IDO) and prostaglandin E2 (PGE2), and showed enhanced in vivo persistence after transplantation. In mouse models of acute colitis and acetaminophen‐induced liver injury, poly‐Z‐cultured hADSC spheroids markedly alleviated inflammation, reduced tissue damage, and modulated pro‐ and anti‐inflammatory cytokine levels more effectively than ULA or TCP‐derived cells. These findings establish the poly‐Z matrix as a potent 3D culture platform that enhances the functional and therapeutic potential of hADSCs for clinical applications.

## Graphical abstract

The 3D culture of human adipose‐derived stem cells (hADSCs) on a crosslinked cyclosiloxane‐based poly‐Z matrix platform enables the formation of spheroid structures enriched with extracellular matrix (ECM). These ECM‐enriched hADSC spheroids cultured on poly‐Z exhibit enhanced differentiation potential, immunomodulatory capacity, and in vivo persistence, collectively resulting in superior therapeutic efficacy in inflammatory disease models.

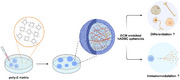

## 1 Introduction

Human mesenchymal stem cells (hMSCs) are adult stem cells that can be isolated from various tissues, including bone marrow, adipose tissue, and umbilical cord blood [1, 2, 3]. Owing to their multipotent differentiation capacity and immunomodulatory properties, hMSCs have garnered significant attention as promising therapeutic agents in regenerative medicine and cell therapy [3, 4, 5, 6, 7]. Among them, human adipose‐derived stem cells (hADSCs) are particularly attractive due to their abundance, ease of harvest through minimally invasive procedures, and low immunogenicity [8, 9, 10]. Compared to hMSCs derived from other tissues, hADSCs demonstrate higher in vitro proliferation rates while maintaining genetic stability during expansion [11]. They also exhibit robust multilineage differentiation potential and high plasticity [12, 13, 14]. In addition, hADSCs secrete a variety of immunomodulatory factors that enable them to modulate the surrounding immune microenvironment [6, 10]. These properties make hADSCs a compelling cell source for regenerative and anti‐inflammatory therapies. Indeed, hADSCs are being actively investigated in preclinical and clinical studies targeting musculoskeletal disorders, inflammatory conditions, and autoimmune diseases [15, 16, 17, 18].

A key prerequisite for the clinical application of hADSCs is the reliable and scalable production of high‐quality cells [17, 19, 20]. Traditionally, hADSCs have been expanded under 2D adherent culture conditions. However, accumulating evidence suggests that prolonged 2D culture can lead to chromosomal abnormalities and cellular senescence, thereby reducing differentiation potential and impairing immunomodulatory function [21, 22, 23, 24]. Consequently, 2D‐cultured hADSCs often exhibit poor engraftment and limited survival upon in vivo transplantation, ultimately restricting their therapeutic efficacy [25, 26, 27]. To address these limitations, a number of strategies have been explored to preserve the differentiation capacity and immunomodulatory function of hADSCs during in vitro expansion. Among them, 3D culture systems have emerged as promising alternatives, as they better recapitulate the in vivo microenvironment, particularly with regard to cell‐cell communication and interactions with the extracellular matrix (ECM) [28, 29, 30, 31]. Various 3D culture platforms, including spheroid cultures, bioreactor systems, and hydrogel scaffolds, have been developed and shown to enhance the expression of stemness‐associated markers such as *OCT4*, *SOX2*, and *NANOG*, supporting the maintenance of multipotency in hADSCs [28, 32, 33]. Moreover, hADSC spheroids exhibit enhanced production of immunomodulatory factors such as PGE2, IDO, TSG‐6, and IL‐10, contributing to improved anti‐inflammatory properties [34, 35]. These findings highlight the potential of hADSC spheroids for therapeutic applications in regenerative medicine.

In our previous studies, we developed a spheroid culture platform based on crosslinked cyclosiloxane polymer films for both cancer cells and human induced pluripotent stem cells (hiPSCs) [36, 37]. Cancer cell spheroids generated on this platform exhibited enhanced self‐renewal, ECM production, drug resistance, and metastatic potential—hallmarks of cancer stem cells [36]. Additionally, hiPSC spheroids cultured on a specific crosslinked cyclosiloxane polymer, designated poly‐Z, maintained long‐term pluripotency in a naïve‐like state, outperforming conventional 2D colonies or suspension‐cultured spheroids [37]. Building on these findings, we hypothesized that the poly‐Z matrix could similarly support hADSC spheroid formation in a manner that enriches ECM components and mimics in vivo‐like conditions, thereby enhancing stemness, differentiation potential, and immunomodulatory activity. In this study, we demonstrate that the spheroid culture of hADSCs on the poly‐Z matrix significantly enhances their differentiation capacity and immunomodulatory functions. We systematically compare the biological and functional properties of poly‐Z‐cultured hADSC spheroids with those produced on conventional 2D culture plates and a commercial spheroid culture system. Furthermore, we assess the therapeutic efficacy of poly‐Z‐cultured hADSC spheroids in two mouse models of inflammatory disease.

## 2 Results

### 2.1 Spheroid Formation of hADSCs on a Poly‐Z Matrix

**FIGURE 1:**
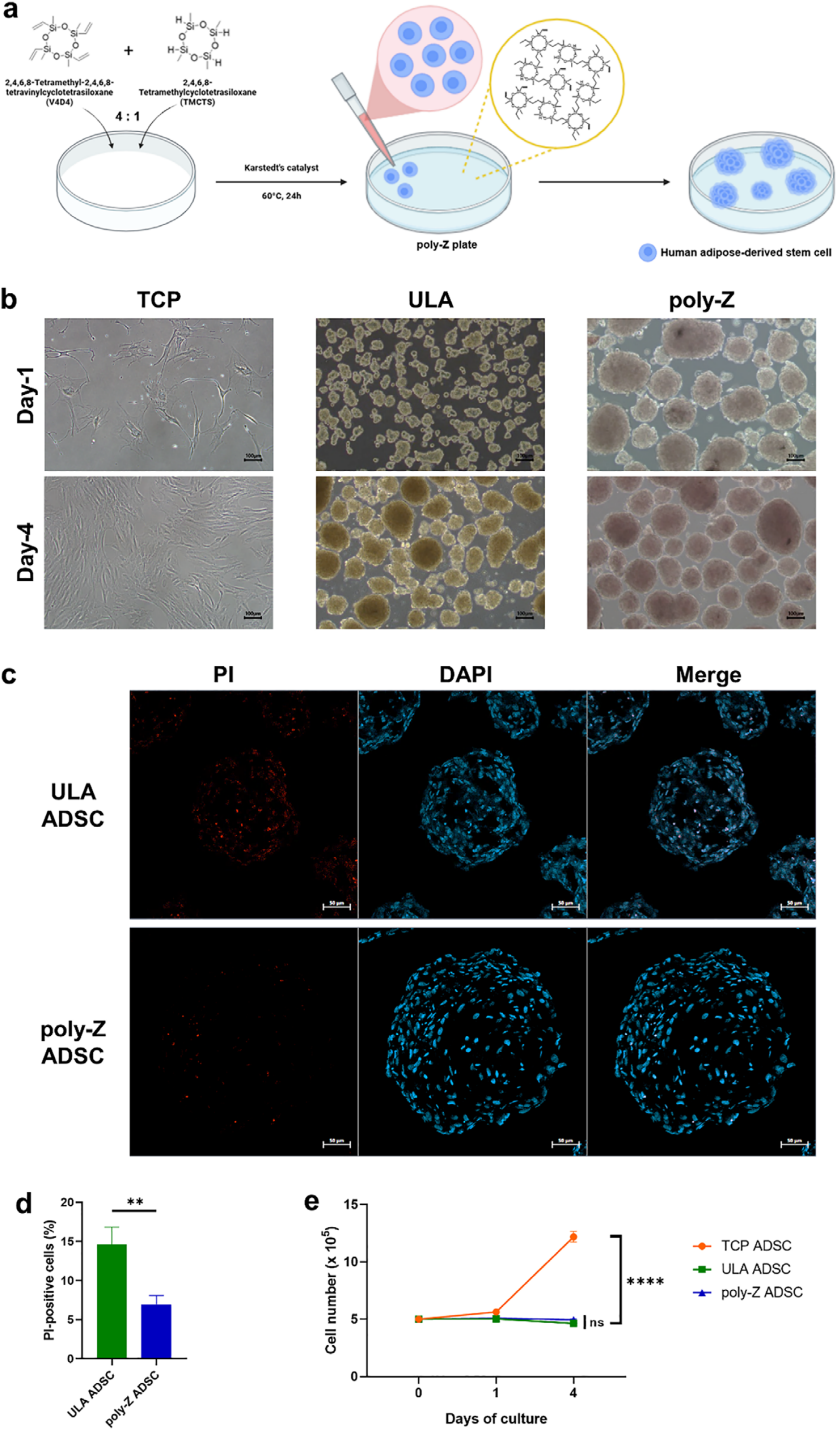
**Spheroid formation of hADSCs on the poly‐Z matrix**. (a) Schematic illustration for the formation of hADSC spheroids on the poly‐Z plate synthesized from crosslinking of two monomers V4D4 and TMCTS in a 4:1 ratio. (b) Representative phase‐contrast images of hADSCs cultured for 1 and 4 days on TCP, ULA, and poly‐Z matrices (5 × 10^4^ cells/cm^2^). (c) Fluorescence images of live/dead staining of hADSC spheroids after 4 days of culture on ULA and poly‐Z. Live cells were stained with DAPI (blue), and dead cells with PI (red). (d) Quantification of PI‐positive (dead) cells within hADSC spheroids cultured on ULA and poly‐Z. (e) Cell proliferation of hADSCs after 1 and 4 days of culture on TCP, ULA, and poly‐Z. Scale bars: 100 µm (b), 50 µm (c). All experiments were performed in triplicate. Data in (d) and (e) are presented as mean ± SD from three independent experiments (n = 3). (ns: not significant; ^**^ *p* < 0.01; ^****^ *p* < 0.0001). (Illustration was created using BioRender.com).

The poly‐Z matrix was prepared by crosslinking two monomers, V4D4 and TMCTS, at a 4:1 ratio on conventional tissue culture plates (TCPs), as previously described [37]. Fourier transform infrared (FT‐IR) analysis confirmed successful polymer formation, as indicated by a marked reduction of the characteristic vinyl bond peaks at approximately 1400 and 1600 cm^−^^1^ (Figure S1). In our previous study, we showed that the stemness of cancer cell spheroids was markedly enhanced when cultured in serum replacement (SR)–supplemented medium compared with medium containing 10% fetal bovine serum (FBS) [36]. Based on this finding, we first examined the effect of medium composition—SR vs. FBS supplementation—on the formation of hADSC spheroids on the poly‐Z matrix (Figure 1a). Conventional TCPs and ultra‐low attachment (ULA) plates were used as controls. After 24 h of culture, spheroid‐like aggregates were observed under both conditions; however, cells cultured in SR‐supplemented medium formed spheroids with larger size and denser morphology than those cultured in FBS‐supplemented medium (Figure S2a). Consistently, SR‐cultured spheroids exhibited significantly higher expression of self‐renewal–associated genes, including *OCT4*, *SOX2*, and *NANOG*, compared with FBS‐cultured spheroids (Figure S2b). In contrast, when hADSCs were cultured on conventional TCPs for 24 h, spheroid formation was not observed in either FBS‐ or SR‐supplemented media (Figure S3). On ULA plates, hADSCs formed spheroids in both culture conditions (Figure S4a). By day 4 of culture, spheroids formed in SR‐supplemented medium reached diameters exceeding ∼400 µm, which was substantially larger than those cultured in FBS‐supplemented medium (∼150–300 µm). Such large spheroids may raise concerns regarding potential necrosis within the spheroid core [38]. Nevertheless, only minor differences in the expression of self‐renewal genes were observed between the two groups (Figure S4b,c). Based on these findings, SR‐supplemented medium was selected for hADSC spheroid culture on the poly‐Z matrix, whereas FBS‐supplemented medium was used for cultures on TCP and ULA plates.

We next compared the morphological and biological characteristics of hADSC spheroids generated on the poly‐Z matrix vs. those formed on ULA plates. After 1 day of culture, poly‐Z‐cultured spheroids were substantially larger than their ULA‐cultured counterparts. By day 4, poly‐Z‐cultured spheroids displayed a more uniform size distribution and denser packing, whereas ULA spheroids remained more heterogeneous and loosely packed (Figure 1b). Live/dead staining further revealed that poly‐Z‐cultured spheroids exhibited significantly lower levels of PI‐positive (dead) cells compared to ULA spheroids, indicating improved cell viability and microenvironmental support (Figure 1c,d). In contrast, while hADSCs cultured on TCP showed increased cell numbers after 4 days due to continuous proliferation, total cell numbers in both ULA and poly‐Z spheroids remained relatively constant (Figure 1e), suggesting that spheroid formation may limit proliferation and instead favor maintenance or enhancement of cell function.

### 2.2 Effects of Poly‐Z Surface Properties on hADSC Spheroid Formation

We next investigated key parameters that may contribute to hADSC spheroid formation on the poly‐Z matrix, in comparison with spheroid formation on ULA surfaces. First, the surface properties of poly‐Z were compared with those of TCP and ULA plates. Atomic force microscopy (AFM) measurements revealed that the poly‐Z–coated surface was considerably smoother and more uniform than those of TCP and ULA plates (Figure S5). In addition, the poly‐Z surface exhibited a water contact angle of ∼101°, indicating a highly hydrophobic surface, whereas TCP and ULA plates showed substantially lower contact angles of ∼56° and ∼32°, respectively (Figure S6). Previous studies have reported that highly hydrophobic surfaces facilitate the adsorption of albumin from cell culture media. This adsorbed protein layer can suppress direct cell adhesion to the substrate and instead promote the self‐assembly of cells into aggregates [39, 40]. Based on this mechanism, we hypothesized that albumin adsorption on the hydrophobic poly‐Z surface may play a key role in mediating hADSC spheroid formation. To test this hypothesis, hADSCs were cultured on poly‐Z in serum‐free medium supplemented with varying concentrations of bovine serum albumin (BSA). After 24 h of culture, hADSCs adhered to the poly‐Z surface in the absence of BSA or at a low concentration (1 mg/mL), whereas spheroid formation was observed at BSA concentrations ≥2 mg/mL (Figure S7a). Although the spheroids formed at 2 mg/mL BSA were relatively small, larger and more numerous spheroids were clearly observed at BSA concentrations ≥4 mg/mL. Quantification of spheroid formation efficiency based on DNA content further confirmed the critical BSA concentration required for hADSC spheroid formation on poly‐Z (Figure S7b). In contrast, ULA plates did not exhibit BSA concentration–dependent behavior and supported spheroid formation across all tested BSA concentrations (Figure S7c,d). These results suggest that spheroid formation on poly‐Z is mediated and facilitated by BSA adsorption, whereas ULA plates promote spheroid formation through a BSA‐independent mechanism. Consistent with this observation, the mRNA expression of *SPARC*, a known albumin‐binding receptor, was markedly upregulated in hADSC spheroids cultured on poly‐Z compared with those cultured on TCP or ULA plates (Figure S7e).

To further verify the role of albumin adsorption in spheroid formation, poly‐Z surfaces were pre‐coated with BSA for 24 h prior to hADSC seeding in serum‐free medium. Similar to the results obtained with albumin‐supplemented medium (Figure S7a,b), surfaces pre‐coated with ≥2 mg/mL BSA promoted spheroid formation even in the absence of BSA in the culture medium, showing a clear BSA concentration–dependent trend (Figure S8a,b). Collectively, these results indicate that spheroid formation of hADSCs on the poly‐Z matrix is mediated by adsorption of BSA onto the hydrophobic surface, which suppresses cell–substrate adhesion and promotes cell self‐assembly into spheroids.

Furthermore, we compared the spheroid‐forming ability of poly‐Z with six poly‐Z analogues synthesized by crosslinking V4D4 and TMCTS at different molar ratios ranging from 4:1 to 1:4 (Figure S9). All poly‐Z analogues supported spheroid formation and showed markedly increased expression of self‐renewal genes compared with hADSCs cultured on TCP. However, the original poly‐Z composition exhibited the most consistent performance in terms of spheroid morphology and gene expression. This observation suggests that the poly‐Z analogues possess similar surface chemical functionalities, likely resulting in comparable levels of albumin adsorption. In addition, varying the thickness of the poly‐Z coating (0.1–0.2 mm) did not significantly affect spheroid‐forming ability or the expression of self‐renewal genes in hADSC spheroids (Figure S10).

### 2.3 Unique Phenotypic Features of Poly‐Z‐Cultured hADSC Spheroids

**FIGURE 2:**
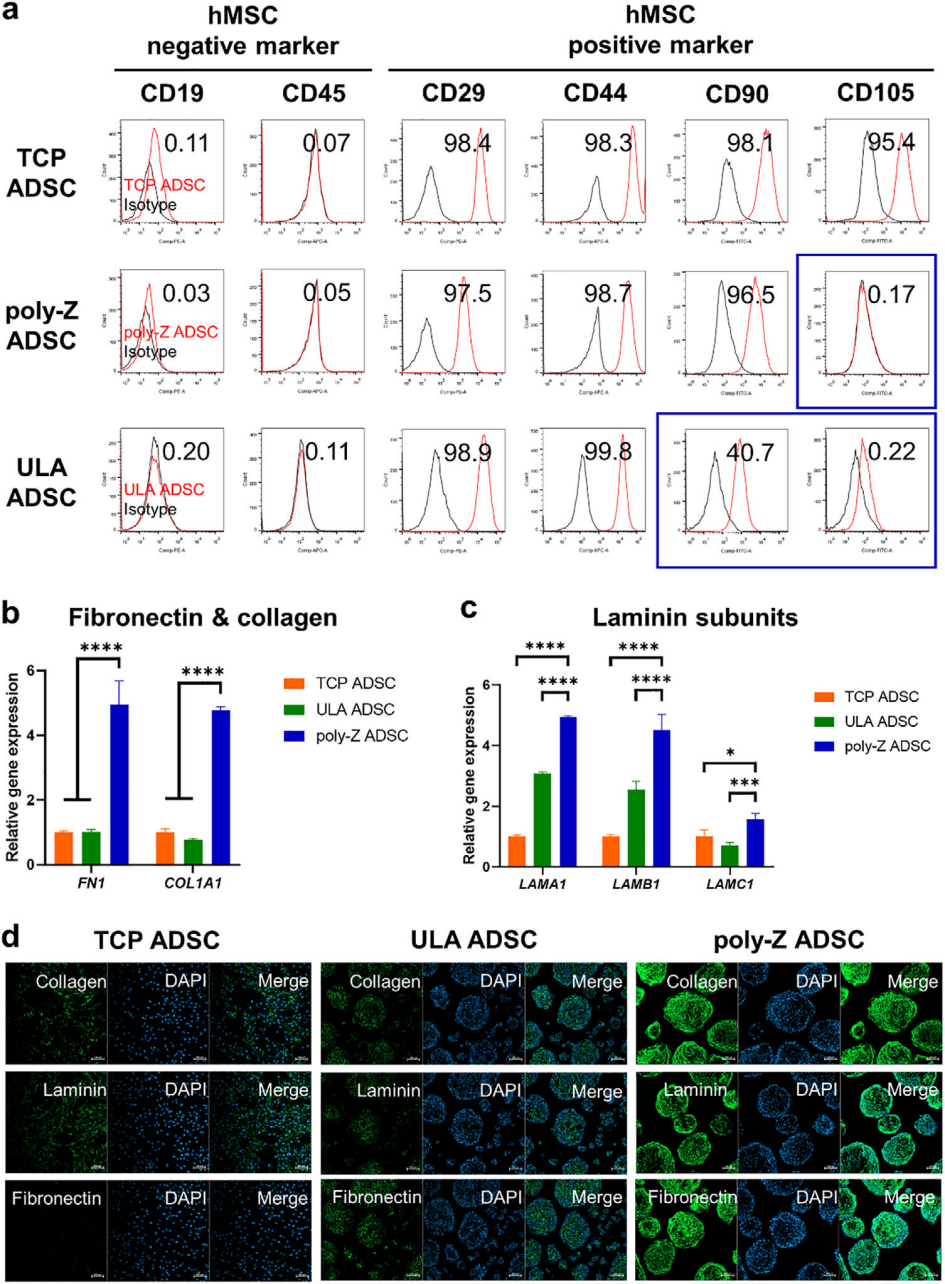
**Phenotypic and extracellular matrix characterization of hADSCs cultured on TCP, ULA, and poly‐Z**. (a) Flow cytometry analysis of hMSC‐negative (CD19, CD45) and hMSC‐positive (CD29, CD44, CD90, CD105) surface markers in hADSCs cultured for 4 days on TCP, ULA, and poly‐Z. (b,c) Relative mRNA expression levels of ECM‐related genes in hADSCs cultured for 4 days: (b) fibronectin (*FN1*) and collagen type I (*COL1A1*), and (c) laminin subunits (*LAMA1*, *LAMB1*, *LAMC1*). (d) Immunocytochemistry of representative ECM proteins (fibronectin, collagen I, and laminin) in hADSCs cultured on TCP, ULA, and poly‐Z. Scale bar: 100 µm (d). All experiments were performed in triplicate. Data in (b) and (c) are presented as mean ± SD from three independent experiments (n = 3). (ns: not significant; ^*^ *p* < 0.05; ^***^ *p* < 0.001; ^****^ *p* < 0.0001).

To investigate phenotypic differences, we analyzed the expression of six surface markers—including two negative (CD19, CD45) and four positive (CD29, CD44, CD90, CD105) mesenchymal stem cell (MSC) markers—in hADSCs cultured for 4 days on TCP, ULA, or poly‐Z matrices. As expected, TCP‐cultured hADSCs lacked expression of hematopoietic markers CD19 and CD45, while strongly expressing MSC‐associated markers CD29, CD44, CD90, and CD105, confirming their typical MSC identity (Figure 2a). Interestingly, CD105 expression was completely absent in both poly‐Z‐ and ULA‐cultured hADSC spheroids, while CD90 expression was markedly reduced only in ULA spheroids (Figure 2a). These observations are notable, as CD105‐negative MSCs have been reported to exhibit enhanced immunomodulatory functions compared to their CD105‐positive counterparts [41, 42]. However, the substantial reduction of CD90 expression—a key MSC marker associated with stemness and therapeutic efficacy—in ULA spheroids may reflect a loss of desirable MSC characteristics, which was not observed in poly‐Z‐cultured hADSC spheroids [43].

To further characterize the extracellular niche, we analyzed the expression of key extracellular matrix (ECM) genes known to be abundant in adipose‐derived tissues, including those encoding fibronectin (*FN1*), type I collagen (*COL1A1*), and laminin subunits (*LAMA1*, *LAMB1*, *LAMC1*) [44]. Quantitative PCR revealed that poly‐Z‐cultured hADSC spheroids exhibited significantly elevated expression of *FN1* (4.9‐fold), *COL1A1* (4.7‐fold), *LAMA1* (4.9‐fold), *LAMB1* (4.5‐fold), and *LAMC1* (1.5‐fold), compared to TCP‐cultured adherent hADSCs (Figure 2b,c). In contrast, ULA‐cultured spheroids showed only marginal or no increase in these ECM genes relative to TCP‐cultured cells. These findings were corroborated by immunocytochemistry (ICC), which showed markedly higher levels of collagen, laminin, and fibronectin proteins in poly‐Z spheroids compared to those in TCP or ULA cultures (Figure 2d). Taken together, these results demonstrate that poly‐Z‐cultured hADSC spheroids acquire a distinct and therapeutically favorable phenotype, characterized by loss of CD105, retention of CD90, and enhanced ECM‐producing capacity—properties not observed in ULA spheroids.

### 2.4 Enhanced Differentiation Capabilities of Poly‐Z‐Cultured hADSC Spheroids

**FIGURE 3:**
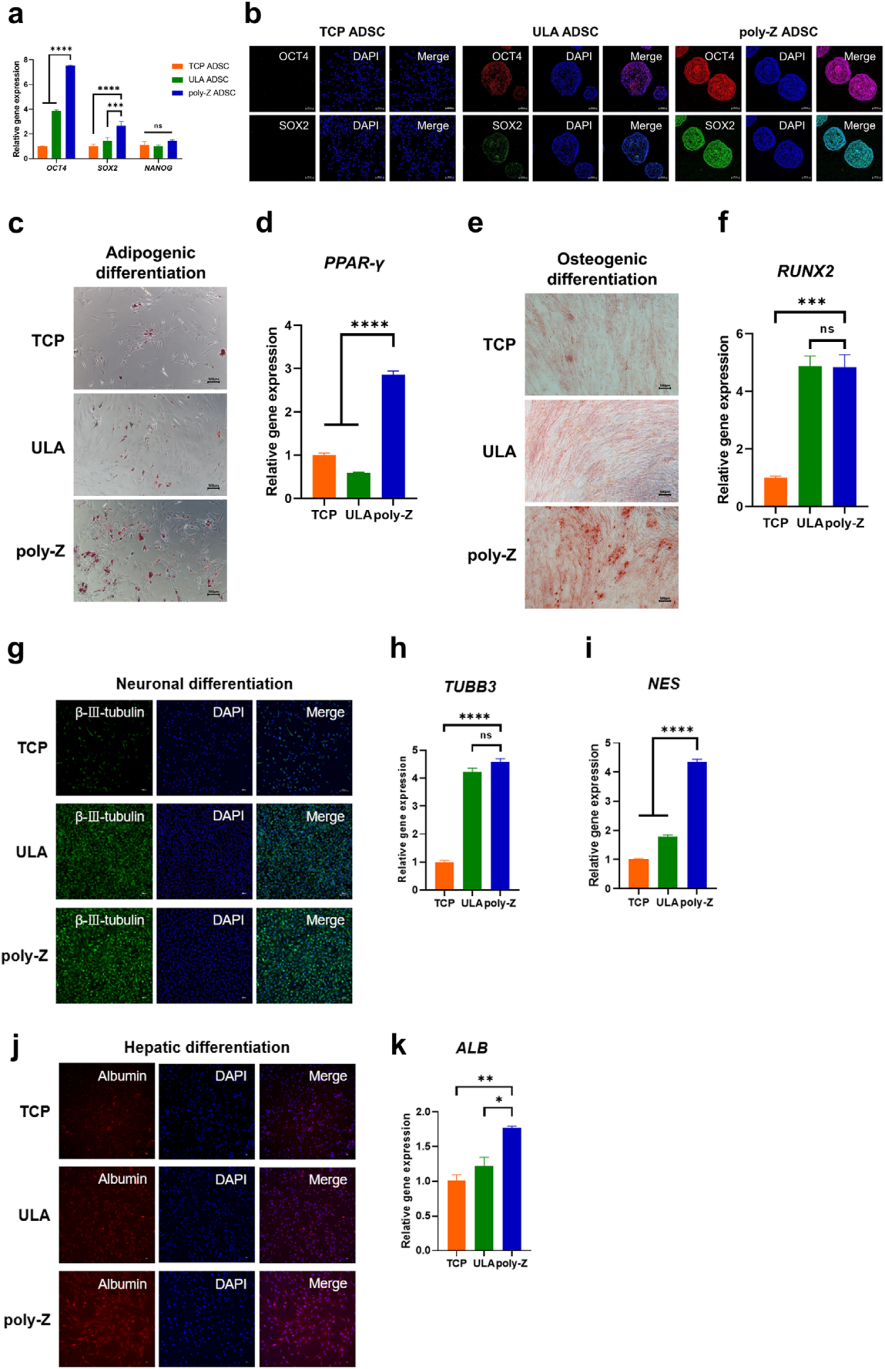
**Expression of pluripotency markers and multilineage differentiation potential of hADSCs cultured on TCP, ULA, and poly‐Z**. (a) Relative mRNA expression of pluripotency‐associated genes (*OCT4*, *SOX2*, *NANOG*) in hADSCs cultured for 4 days on TCP, ULA, and poly‐Z. (b) Immunocytochemistry (ICC) for pluripotency markers (OCT4 and SOX2) in hADSCs cultured under the same conditions. (c,d) Assessment of adipogenic differentiation: (c) Oil Red O staining of lipid droplets, and (d) relative expression of the adipogenic marker gene *PPAR‐γ*. (e,f) Assessment of osteogenic differentiation: (e) Alizarin Red S staining for calcium deposits, and (f) relative expression of the osteogenic marker gene *RUNX2*. (g–i) Assessment of neuronal transdifferentiation: (g) ICC for the neuronal marker β‐III‐tubulin, (h) expression of the neuronal marker gene *TUBB3*, and (i) expression of the neuronal marker gene *NES*. (j,k) Assessment of hepatic transdifferentiation: (j) ICC for the hepatic marker albumin, and (k) expression of the hepatic marker gene *ALB*. Scale bars: 100 µm (b,c,e,g,j). All experiments were performed in triplicate. Data in (a,d,f,h,i,k) are presented as mean ± SD from three independent experiments (n = 3). (ns: not significant; ^*^ *p* < 0.05; ^**^ *p* < 0.01; ^***^ *p* < 0.001; ^****^ *p* < 0.0001). Representative images are shown (c–k); quantification was performed from multiple randomly selected fields across three independent experiments.

Having confirmed the enrichment of extracellular matrix (ECM) components in poly‐Z‐cultured hADSC spheroids, we next investigated their effects on the expression of pluripotency markers. Notably, hADSC spheroids cultured on poly‐Z exhibited significantly increased expression of *OCT4* (7.5‐fold) and *SOX2* (2.6‐fold) compared to TCP‐cultured adherent hADSCs. These increases were also markedly higher than those observed in ULA‐cultured spheroids, which showed 3.8‐fold and 1.5‐fold increases in *OCT4* and *SOX2*, respectively (Figure 3a). In contrast, the expression of *NANOG* remained unchanged across all three groups. These gene expression findings were corroborated by immunocytochemistry (ICC), which revealed the highest protein levels of OCT4 and SOX2 in poly‐Z‐cultured spheroids (Figure 3b). These results suggest that spheroid formation on the poly‐Z matrix supports enhanced stemness and may potentiate the differentiation capacity of hADSCs.

To evaluate the mesodermal differentiation potential, hADSC spheroids from all groups were enzymatically dissociated into single cells and subjected to adipogenic and osteogenic differentiation using lineage‐specific induction media. Differentiation efficiency was assessed through lineage‐specific staining and marker gene expression. Upon adipogenic induction, poly‐Z‐cultured cells exhibited more prominent lipid droplet formation and significantly higher expression of *PPAR‐γ*, a key adipocyte marker, compared to TCP‐ and ULA‐derived cells (Figure 3c,d). Similarly, osteogenic induction resulted in greater calcium deposition and higher *RUNX2* expression in both ULA‐ and poly‐Z–cultured spheroids than TCP controls (Figure 3e,f), with poly‐Z spheroids showing a slight edge over ULA.

To further assess trans‐differentiation potential toward ectodermal and endodermal lineages, single‐cell suspensions derived from hADSC spheroids were induced to undergo neuronal and hepatic differentiation. Following neuronal induction, β‐III‐tubulin staining and its corresponding gene expression were both higher in the ULA and poly‐Z groups than in the TCP group (Figure 3g,h). In addition, the gene expression of *NES*, another neuronal marker, was significantly higher in poly‐Z‐derived cells than in those from the TCP and ULA groups (Figure 3i). Following hepatic induction, poly‐Z‐derived cells displayed a higher proportion of albumin‐positive cells (Figure 3j) and significantly elevated *ALB* gene expression (Figure 3k). Although ULA‐cultured hADSC spheroids showed improved neuronal and hepatic marker expression relative to TCP‐cultured cells, their differentiation efficiency into hepatocytes remained lower than that of poly‐Z‐cultured spheroids. Collectively, these results demonstrate that hADSC spheroids cultured on the poly‐Z matrix exhibit markedly enhanced mesodermal differentiation as well as ectodermal and endodermal trans‐differentiation capacity, outperforming both 2D (TCP) and conventional spheroid (ULA) culture systems. These findings underscore the superior potential of the poly‐Z platform for promoting multilineage differentiation of hADSCs.

### 2.5 Unique Immunomodulatory Profiles of Poly‐Z‐Cultured hADSC Spheroids

**FIGURE 4:**
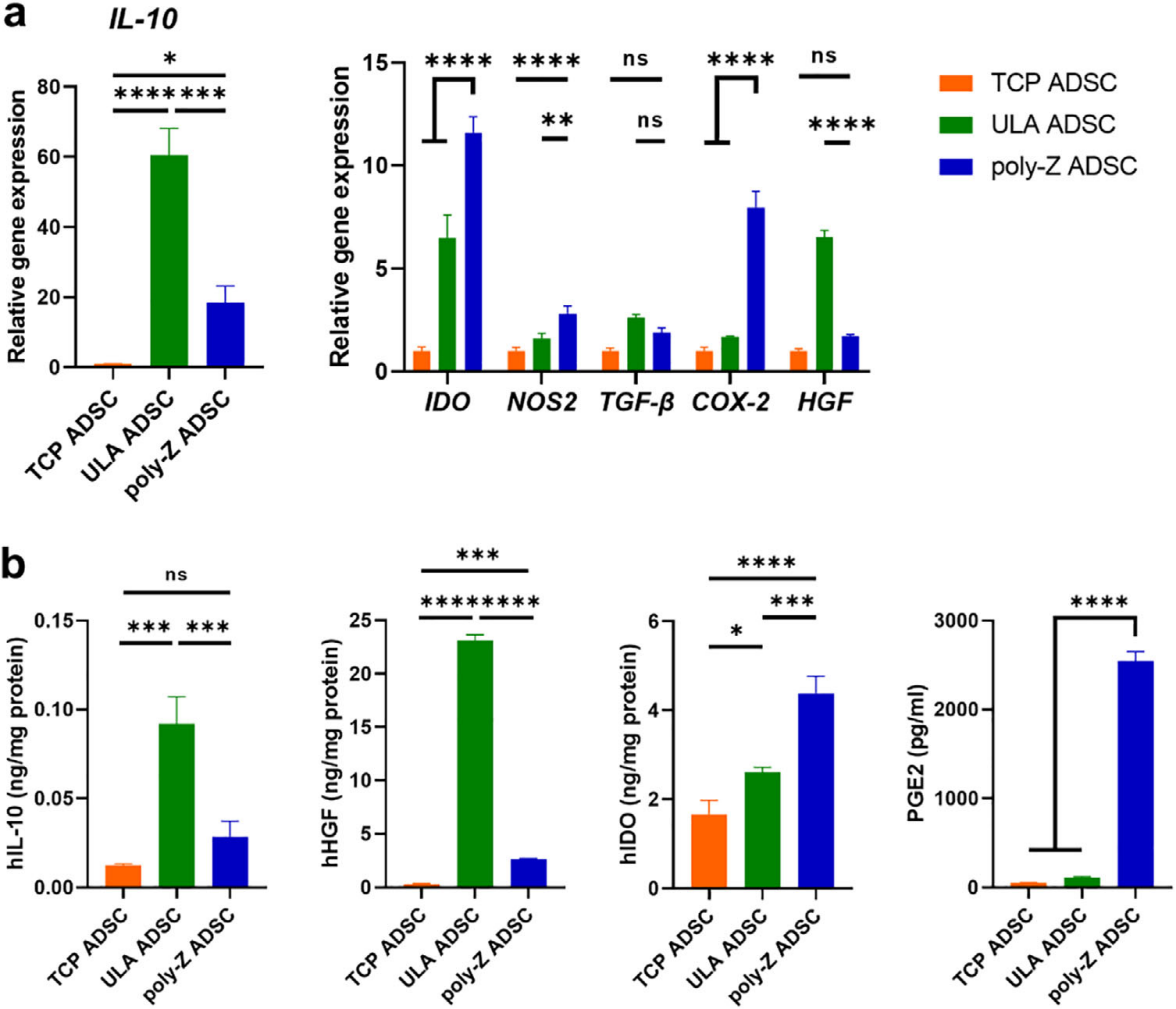
**Expression and secretion of immunomodulatory factors in hADSCs cultured on TCP, ULA, and poly‐Z**. (a) Relative mRNA expression levels of immunomodulatory genes (*IL‐10*, *IDO*, *NOS2*, *TGF‐β*, *COX‐2*, and *HGF*) in hADSCs cultured for 4 days on TCP, ULA, and poly‐Z. (b) Quantification of secreted immunomodulatory proteins (hIL‐10, hHGF, hIDO, and PGE2) in serum‐free conditioned media collected after 24 h from hADSCs pre‐cultured for 4 days on each substrate. All experiments were performed in triplicate. Data in (a) and (b) are presented as mean ± SD from three independent experiments (n = 3). (ns: not significant; ^*^ *p* < 0.05; ^**^ *p* < 0.01; ^***^ *p* < 0.001; ^****^ *p* < 0.0001).

Given the critical role of immunomodulatory functions in the therapeutic efficacy of hADSCs, we next analyzed the expression and secretion of key immunomodulatory factors. After 4 days of culture, both ULA‐ and poly‐Z‐derived hADSC spheroids exhibited markedly elevated expression of canonical immunomodulatory genes—including *IL‐10*, *IDO*, *NOS2*, *TGF‐β*, *COX‐2*, and *HGF*—compared to adherent TCP‐cultured hADSCs (Figure 4a). Interestingly, gene expression patterns revealed distinct immunomodulatory signatures between the two spheroid culture systems. ULA‐cultured spheroids showed higher expression of *IL‐10*, *TGF‐β*, and *HGF*, while poly‐Z‐cultured spheroids expressed higher levels of *IDO*, *NOS2*, and *COX‐2* (Figure 4a). This divergence suggests that while both 3D systems enhance immunomodulatory gene expression relative to 2D culture, the underlying mechanisms and functional outputs may differ.

To validate these findings at the protein level, we conducted ELISA on cell culture supernatants. Consistent with gene expression results, poly‐Z‐cultured spheroids secreted significantly greater amounts of hIDO and PGE2 (a downstream product of COX‐2), whereas ULA‐cultured spheroids released higher levels of hIL‐10 and hHGF (Figure 4b). These data confirm that both spheroid systems enhance the immunoregulatory potential of hADSCs, but poly‐Z‐cultured spheroids exhibit a distinct profile skewed toward IDO‐ and PGE2‐mediated immunosuppression, whereas ULA spheroids favor IL‐10 and HGF‐mediated pathways. Taken together, these findings highlight that 3D culture—whether on ULA or poly‐Z substrates—significantly enhances the immunomodulatory function of hADSCs relative to conventional 2D culture. Notably, poly‐Z‐cultured spheroids display a unique immunomodulatory signature, which may confer distinct therapeutic advantages, particularly in inflammatory disease models where specific cytokine and enzyme pathways are more critical.

### 2.6 Effects of Cell‐ECM Interactions on the Enhanced Stemness and Immunomodulatory Functions of Poly‐Z‐Cultured hADSC Spheroids

**FIGURE 5:**
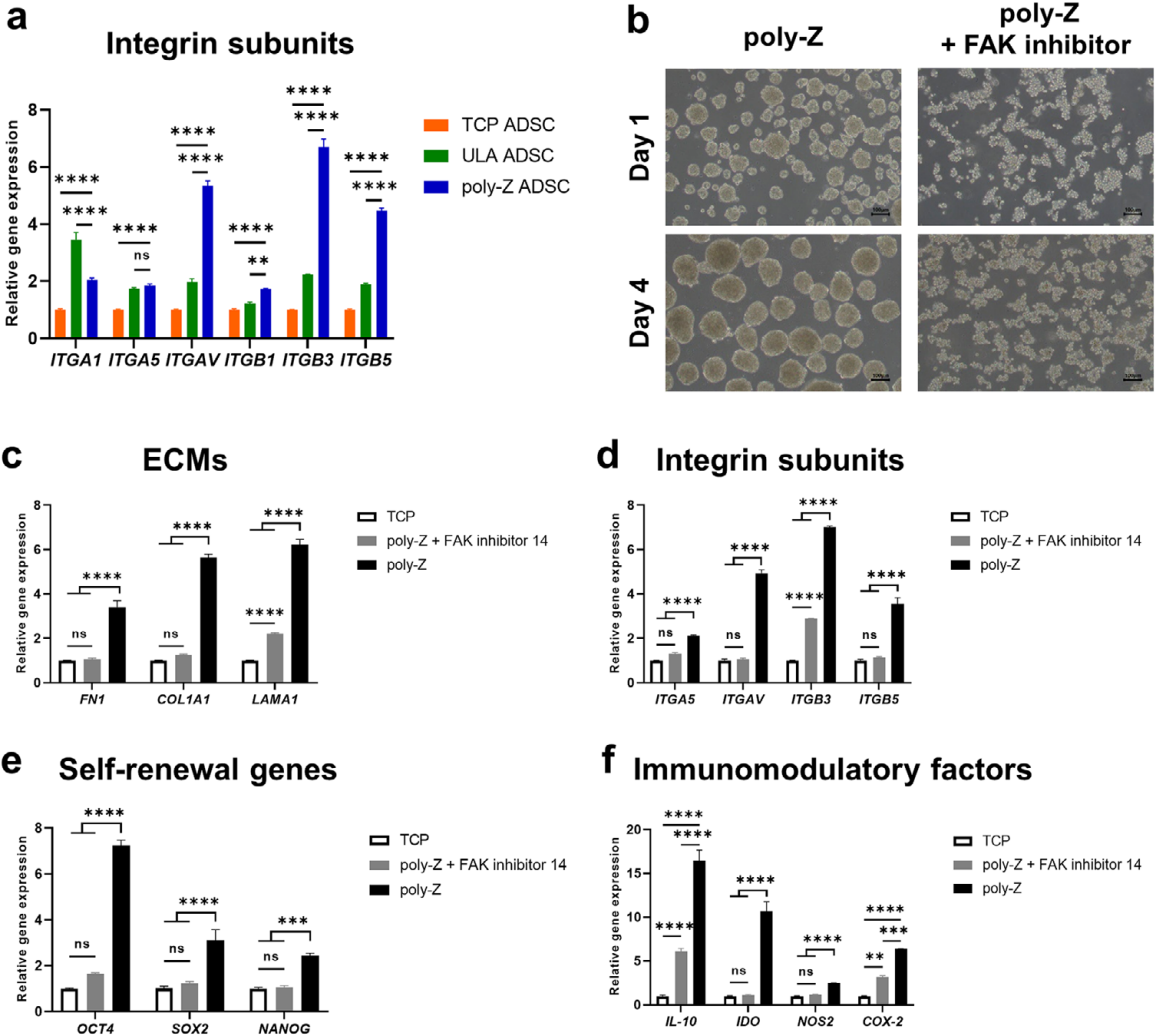
**FAK signaling mediates the enhanced stemness and immunomodulatory functions of poly‐Z–derived hADSC spheroids**. (a) Relative mRNA expression of integrin subunits (*ITGA1, ITGA5, ITGAV, ITGB1, ITGB3*, and *ITGB5*) in hADSCs cultured on TCP, ULA, and poly‐Z for 4 days. (b) Representative morphologies of hADSCs cultured on poly‐Z for 1 and 4 days in the absence or presence of FAK inhibitor 14 (10 µm). (c–f) Relative mRNA expression of (c) ECM‐related genes, (d) integrin subunits, (e) pluripotency‐associated genes, and (f) immunomodulatory genes in hADSCs cultured on TCP or poly‐Z for 4 days with or without FAK inhibitor 14. Scale bar: 100 µm (b). All experiments were performed in triplicate. Data in (a,c–f) are presented as mean ± SD from three independent experiments (n = 3). (ns: not significant; ^*^ *p* < 0.05; ^***^ *p* < 0.001; ^****^ *p* < 0.0001).

Since interactions between cells and ECM components are known to regulate the survival, migration, differentiation, and regenerative potential of hMSCs [45, 46, 47], we investigated whether cell–ECM interactions contribute to the enhanced differentiation potential and immunomodulatory phenotypes observed in poly‐Z‐cultured hADSC spheroids. Given that poly‐Z‐cultured spheroids exhibited pronounced ECM enrichment (Figure 2b–d), we first examined the expression of integrins. As expected, hADSC spheroids cultured on both ULA and poly‐Z surfaces showed higher integrin expression than 2D‐cultured hADSCs on TCP (Figure 5a). Notably, however, with the exception of integrin α1 and α5, all other integrin subunits were significantly more upregulated in poly‐Z‐cultured spheroids than in ULA‐cultured spheroids (Figure 5a). Among these, integrin α_V_β_3_—which is known to interact with ECM components and activate focal adhesion kinase (FAK) signaling to regulate hMSC stemness and immunomodulatory functions—was markedly upregulated [48, 49, 50]. Based on this observation, we next investigated the effects of FAK inhibition on spheroid formation, stemness, and immunomodulatory activity of hADSCs cultured on poly‐Z. In the absence of a FAK inhibitor, hADSCs formed compact spheroids on the poly‐Z matrix within 24 h. In contrast, when FAK signaling was inhibited, hADSCs failed to form spheroids even after 4 days of culture and instead formed grape cluster‐like cell aggregates (Figure 5b). We then examined the expression of ECM components, integrins, self‐renewal genes, and immunomodulatory factors in hADSCs cultured on poly‐Z for 4 days in the absence or presence of the FAK inhibitor. Under FAK inhibition, the expression of multiple ECM‐related genes and integrins was markedly reduced to levels comparable to those observed in TCP‐cultured hADSCs (Figure 5c,d). In addition, the enhanced expression of self‐renewal genes observed in poly‐Z‐cultured hADSC spheroids was significantly attenuated upon FAK inhibition (Figure 5e). Similarly, the expression levels of all examined immunomodulatory factors were substantially diminished compared with those observed in the absence of FAK inhibition (Figure 5f). Collectively, these results suggest that activation of FAK signaling, mediated by interactions between integrins—particularly integrin α_V_β_3_—and ECM components, plays a critical role in promoting the enhanced stemness and immunomodulatory activity of hADSC spheroids formed on the poly‐Z matrix.

### 2.7 Therapeutic Effects of Poly‐Z‐Cultured hADSC Spheroids in a Mouse Model of Acute Colitis

**FIGURE 6:**
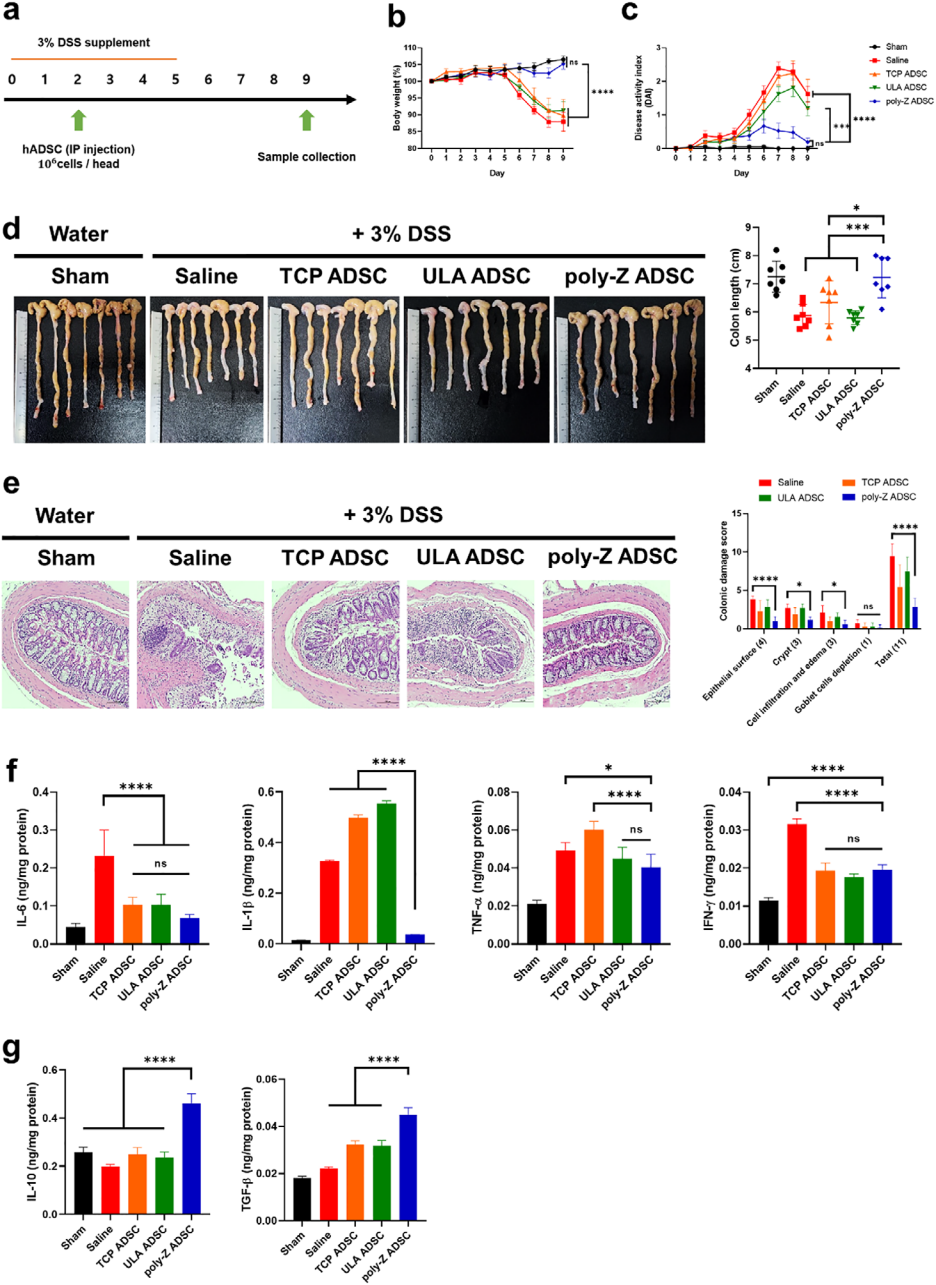
**Therapeutic efficacy of poly‐Z–derived hADSC spheroids in a DSS‐induced acute colitis mouse model**. (a) Experimental timeline of DSS administration and hADSC treatment. Colitis was induced by providing 3% DSS in drinking water for 5 days. On day 2 of DSS treatment, mice were intraperitoneally injected with TCP‐cultured hADSCs or hADSC spheroids derived from ULA or poly‐Z cultures. All mice were sacrificed on day 9. (b–d) Evaluation of disease progression: (b) body weight changes, (c) disease activity index (DAI), and (d) final colon lengths. (e) Representative histological images of colon tissue stained with hematoxylin and eosin (H&E). (f) Quantification of pro‐inflammatory cytokines (IL‐6, IL‐1β, TNF‐α, and IFN‐γ) in distal colon tissue. (g) Quantification of anti‐inflammatory cytokines (IL‐10 and TGF‐β) in distal colon tissue. Scale bar: 100 µm (e). All results are presented as mean ± SD from seven independent biological replicates (n = 7 per group). (ns: not significant; ^*^ *p* < 0.05; ^***^ *p* < 0.001; ^****^ *p* < 0.0001).

Having established the enhanced immunomodulatory profiles of poly‐Z‐cultured hADSC spheroids at both the gene and protein levels, we next investigated their therapeutic efficacy in a dextran sulfate sodium (DSS)‐induced acute colitis mouse model. Colitis was induced by administering 3% DSS in drinking water for 5 days. On day 2, mice were intraperitoneally injected with TCP‐cultured hADSCs or ULA‐ or poly‐Z–cultured hADSC spheroids, each mixed with the synthetic hydrogel Vitrogel (Figure 6a). Therapeutic outcomes were assessed through daily monitoring of body weight and disease activity index (DAI). Mice treated with poly‐Z‐cultured hADSC spheroids exhibited significantly improved clinical parameters, including reduced body weight loss and lower DAI scores, compared to all other treatment groups (Figure 6b,c). In contrast, mice receiving TCP‐cultured hADSCs or ULA‐cultured spheroids showed no statistically significant improvement relative to the saline‐treated disease control group. Macroscopic analysis further revealed that colon lengths in the poly‐Z spheroid–treated group were comparable to those of healthy controls, while the other treatment groups displayed markedly shortened colons, a hallmark of colitis‐associated inflammation (Figure 6d). These findings were corroborated by histological examination of colon sections, which showed that poly‐Z‐treated mice had substantially reduced tissue damage, including preserved crypt structure, decreased immune cell infiltration, and minimal edema, in stark contrast to the severe pathology observed in the DSS‐only group (Figure 6e).

To assess immunological responses at the molecular level, colon tissue extracts were analyzed by ELISA on day 9. Mice treated with poly‐Z‐cultured spheroids exhibited significantly reduced levels of pro‐inflammatory cytokines—including IL‐6, IL‐1β, TNF‐α, and IFN‐γ—compared with the saline‐treated group (Figure 6f). Notably, although the levels of most inflammatory cytokines did not differ significantly between the ULA‐ and poly‐Z‐derived spheroid groups, IL‐1β was significantly reduced only in the poly‐Z group. Moreover, this group showed markedly elevated levels of anti‐inflammatory cytokines, including IL‐10 and TGF‐β (Figure 6g). In contrast, mice treated with TCP‐cultured hADSCs or ULA spheroids exhibited only modest reductions in pro‐inflammatory cytokines and minimal increases in anti‐inflammatory cytokines, suggesting insufficient therapeutic modulation. Taken together, these results clearly demonstrate that poly‐Z‐cultured hADSC spheroids exhibit superior immunomodulatory and therapeutic efficacy in an acute colitis model compared to both 2D‐cultured hADSCs and ULA‐derived spheroids. These findings highlight the clinical potential of the poly‐Z platform for generating highly functional stem cell therapeutics for inflammatory bowel disease.

### 2.8 Therapeutic Effects of Poly‐Z‐Cultured hADSC Spheroids in a Mouse Model of Acute Liver Injury

**FIGURE 7:**
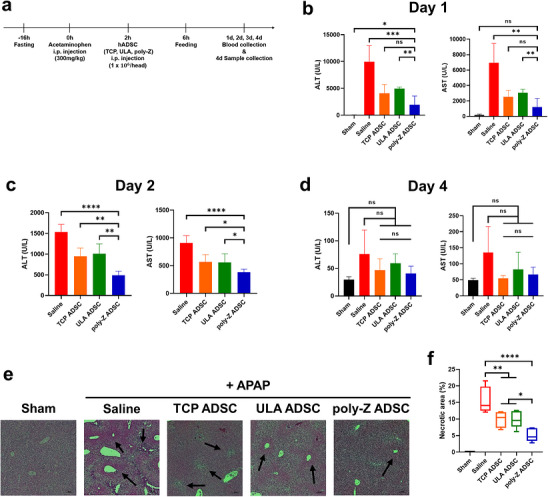
**Therapeutic efficacy of poly‐Z–derived hADSC spheroids in a mouse model of APAP‐induced acute liver injury**. (a) Experimental timeline of acetaminophen (APAP) administration and hADSC treatment. Mice were fasted for 16 h, then intraperitoneally injected with APAP (300 mg/kg) to induce acute liver injury. Two hours later, mice were intraperitoneally injected with TCP‐cultured hADSCs or hADSC spheroids derived from ULA or poly‐Z cultures. Blood samples were collected daily, and mice were sacrificed on day 4. (b–d) Serum levels of liver injury markers: alanine aminotransferase (ALT) and aspartate aminotransferase (AST) on (b) day 1, (c) day 2, and (d) day 4 post‐APAP injection. (e) Representative histological images of liver tissue stained with hematoxylin and eosin (H&E). Zonal necrosis is indicated by dark pink regions (arrows). (f) Quantification of necrotic areas in H&E‐stained tissue sections. Scale bar: 100 µm (e). Data in (b,c,d,f) are presented as mean ± SD from five independent biological replicates (n = 5 per group). (ns: not significant; ^*^ *p* < 0.05; ^**^ *p* < 0.01; ^***^ *p* < 0.001; ^****^ *p* < 0.0001).

To further validate the therapeutic potential of poly‐Z‐cultured hADSC spheroids, we evaluated their efficacy in a mouse model of acetaminophen (APAP)‐induced acute liver injury (ALI). Mice were fasted for 16 h prior to intraperitoneal injection of APAP (300 mg/kg) to induce hepatotoxicity. Two hours post‐APAP administration, mice were intraperitoneally injected with 1 × 10^6^ TCP‐cultured hADSCs or ULA‐ and poly‐Z‐cultured hADSC spheroids premixed with Vitrogel. Food was reintroduced 4 h after injection. Serum samples were collected daily for 4 days to monitor liver injury progression by measuring alanine aminotransferase (ALT) and aspartate aminotransferase (AST) levels (Figure 7a). On day 1, all APAP‐injected groups exhibited a sharp elevation in serum ALT and AST, confirming induction of acute liver damage. Among the treatment groups, mice treated with poly‐Z‐cultured hADSC spheroids displayed the greatest reduction in both ALT and AST levels, compared to the saline‐treated disease group (Figure 7b). By day 2, ALT and AST levels had decreased across all APAP‐treated groups, suggesting partial spontaneous recovery of liver function. Despite this trend, poly‐Z–cultured hADSC spheroids still resulted in significantly lower enzyme levels than those treated with TCP‐ or ULA‐cultured hADSCs (Figure 7c). By day 4, no statistically significant differences in ALT or AST levels were observed among the groups (Figure 7d), likely due to continued spontaneous regeneration of hepatic tissue.

To assess the histopathological outcome, liver tissues collected on day 4 were subjected to H&E staining. Saline‐treated mice exhibited extensive hepatic damage, including widespread necrosis and numerous apoptotic bodies. Similar histological features were observed in the TCP‐ and ULA‐treated groups. In contrast, mice treated with poly‐Z‐cultured hADSC spheroids exhibited markedly reduced necrotic areas and fewer apoptotic cells, indicating more effective amelioration of liver injury (Figure 7e,f). Collectively, these results demonstrate that poly‐Z‐cultured hADSC spheroids provide superior hepatoprotective effects in APAP‐induced ALI compared to hADSCs cultured under conventional 2D or ULA‐based 3D conditions. These findings highlight the potential of the poly‐Z platform to enhance stem cell‐based therapies for acute hepatic inflammation and injury.

### 2.9 In Vivo Retention of Transplanted hADSC Spheroids

To investigate the in vivo persistence of transplanted hADSCs, we evaluated cell retention at the injection site over time using a near‐infrared fluorescence–based tracking approach. hADSCs were labeled with the fluorescent dye DiR, and equal numbers of DiR‐labeled TCP‐cultured hADSCs or ULA‐ and poly‐Z‐cultured hADSC spheroids were subcutaneously injected into the dorsal region of mice. Fluorescence signals were monitored at multiple time points using an in vivo imaging system (IVIS). Up to day 3 post‐injection, all groups exhibited strong and comparable fluorescence signals at the injection site, indicating initial engraftment of transplanted cells (Figure S11). However, by day 5 and especially day 7, the signal from ULA‐cultured hADSC spheroids had nearly disappeared, suggesting poor cell survival or rapid clearance. In contrast, TCP‐ and poly‐Z‐cultured hADSCs retained detectable signals, with the poly‐Z group showing the highest fluorescence intensity, indicating superior in vivo persistence. These findings suggest that poly‐Z‐cultured hADSC spheroids possess enhanced survival and retention capacity in vivo compared to both ULA spheroids and TCP‐cultured cells. This prolonged presence may underlie their superior anti‐inflammatory and therapeutic efficacy observed in mouse models of inflammatory disease.

## 3 Discussion

In this study, we demonstrated that spheroid culture of human adipose‐derived mesenchymal stem cells (hADSCs) using an octagonal cyclosiloxane‐based synthetic polymer matrix, designated poly‐Z, markedly enhanced their stemness, differentiation capacity, and immunomodulatory functions, both in vitro and in vivo. Compared to conventional adherent culture and ultra‐low attachment (ULA)‐based spheroid culture, the poly‐Z platform yielded hADSC spheroids with distinct phenotypic and functional profiles at both gene and protein levels. Our findings underscore the critical importance of microenvironmental cues provided by the cell culture substrate in regulating stem cell fate and function [51]. Although ULA plates are widely used for 3D spheroid formation of hADSCs, our results consistently show that poly‐Z‐cultured hADSC spheroids exhibit superior characteristics compared to those generated on ULA plates. First, cell viability was significantly higher in poly‐Z‐cultured spheroids, as evidenced by a lower proportion of dead cells (Figure 1c,d), suggesting that the poly‐Z matrix offers a more favorable microenvironment for cell survival than the inert, non‐adhesive ULA surface. Second, poly‐Z‐cultured spheroids exhibited substantially higher levels of ECM proteins, including collagen type I, fibronectin, and laminin subunits, than ULA‐cultured spheroids (Figure 2b–d). Given the well‐established role of ECM in mediating cell‐cell and cell‐matrix interactions, this enriched ECM environment may contribute to the maintenance of stemness and enhancement of functional capacity in poly‐Z spheroids [28]. Third, although ULA‐cultured hADSC spheroids also showed increased expression of pluripotency markers and enhanced differentiation potential, the magnitude of these enhancements was consistently greater in poly‐Z‐cultured spheroids (Figure 3). While both ULA‐ and poly‐Z‐cultured spheroids exhibited elevated immunomodulatory gene and protein expression compared to TCP‐cultured cells (Figure 4), their in vivo therapeutic efficacy diverged markedly. In two different models of inflammatory disease (DSS‐induced colitis and APAP‐induced acute liver injury), poly‐Z‐derived spheroids consistently outperformed ULA‐derived and adherent hADSCs in mitigating inflammation, restoring tissue integrity, and modulating cytokine levels (Figures 6 and 7). This functional superiority may be partially attributed to the prolonged in vivo retention of poly‐Z–cultured hADSC spheroids, as shown by fluorescence‐based tracking (Figure S11), highlighting the importance of ECM‐driven in vivo survival for therapeutic outcomes [52]. These findings are in line with prior studies emphasizing that matrix composition and 3D organization significantly influence stem cell behavior, survival, and therapeutic efficacy following transplantation [29, 51, 53].

A particularly noteworthy finding is the differential expression of surface markers, which may underlie functional distinctions between ULA‐ and poly‐Z‐cultured spheroids. While both groups exhibited a complete loss of CD105, only ULA spheroids showed a substantial reduction in CD90 (Figure 2a). CD105 (endoglin) is a co‐receptor for TGF‐β, predominantly expressed on MSCs, and plays a key role in proliferation, differentiation, and immunoregulation [54]. Several studies have reported that CD105‐negative MSCs display enhanced pluripotency gene expression, improved differentiation potential, and increased secretion of immunomodulatory factors such as TGF‐β, supporting the notion that CD105 loss may contribute to enhanced therapeutic efficacy [41, 42]. In contrast, the role of CD90 (Thy‐1) is more complex. CD90 has been implicated in modulating cell signaling, adhesion, and migration, and its expression is thought to be context‐dependent [55]. While some studies suggest that CD90 may promote apoptosis by modulating Fas receptor signaling and suppressing anti‐apoptotic proteins such as Bcl‐2 and Bcl‐xL [56], others report that CD90 enhances cell adhesion and survival by interacting with integrins and activating the FAK/Akt pathway [57, 58]. In 3D spheroid systems where cell‐cell and cell‐ECM interactions are critical, CD90‐mediated signaling likely plays a beneficial role in maintaining cell viability and structural integrity. Therefore, reduced CD90 expression in ULA spheroids may compromise cell adhesion and survival, leading to increased cell death (Figure 1c,d) and poorer in vivo persistence (Figure S11), which could explain their inferior therapeutic efficacy compared to poly‐Z spheroids.

Surface properties of biomaterial substrates—including functional groups, roughness, and hydrophobicity—are known to influence the behavior of human mesenchymal stem cells (hMSCs) [59]. In this study, we focused on surface hydrophobicity as a key determinant regulating hADSC organization and functional enhancement. Hydrophobic surfaces can promote the adsorption of albumin from culture media, generating an interfacial layer that suppresses cell adhesion and facilitates cell self‐assembly into aggregates [39]. Consistent with this mechanism, the poly‐Z matrix exhibited a highly hydrophobic surface, as confirmed by water contact angle measurements (Figure S6), and spheroid formation on poly‐Z depended on albumin supplementation (Figure S7a,b). In contrast, hADSCs cultured on hydrophilic ULA plates formed spheroids regardless of albumin presence (Figure S7c,d), indicating that spheroid formation on poly‐Z is albumin‐mediated, whereas ULA‐induced spheroid formation occurs through an albumin‐independent mechanism. Supporting this interpretation, the expression of *SPARC*, an albumin‐binding receptor, was markedly upregulated in poly‐Z‐cultured spheroids compared with those cultured on TCP or ULA (Figure S7e). SPARC is known to enhance the regenerative capacity of hMSCs and promote the assembly of extracellular matrix (ECM) components such as collagen and fibronectin [60, 61, 62]. Accordingly, poly‐Z spheroids with elevated *SPARC* expression also exhibited increased expression of multiple ECM genes (Figure 2b,c) and integrins interacting with these ECM components (Figure 5a). These results suggest a structure–property–function relationship in which the hydrophobic siloxane‐based surface of poly‐Z promotes serum protein adsorption, thereby modulating cell–material interactions and enabling ECM‐rich spheroid formation.

Further analyses showed that ECM–integrin interactions activated focal adhesion kinase (FAK) signaling, leading to enhanced stemness and immunomodulatory function in poly‐Z‐cultured hADSC spheroids (Figure 5b–f). Together, these findings indicate that the mechanism of spheroid formation can substantially influence the biological properties of resulting spheroids [28], and highlight the important role of albumin‐mediated surface–protein interactions in regulating 3D stem cell culture systems.

Despite the promising findings, this study has several limitations. First, the long‐term fate of transplanted spheroids, including their differentiation potential, functional integration, and possible tumorigenicity, warrants comprehensive investigation to assess safety and translational potential. Second, the lack of proliferative capacity in poly‐Z–cultured hADSC spheroids suggests that this system may not be suitable for large‐scale cell expansion required for clinical applications [17, 19, 20]. However, our results suggest that the poly‐Z matrix supports a unique 3D microenvironment that synergistically enhances both intrinsic properties of stem cells and cell‐ECM interactions, resulting in improved therapeutic functionality. Therefore, this platform could serve as a valuable strategy for ex vivo preconditioning of stem cells before transplantation, especially in clinical applications requiring high immunomodulatory activity and regenerative potential

## 4 Conclusion

In summary, we demonstrate that the synthetic polymer matrix poly‐Z enables robust spheroid formation of hADSCs with markedly enhanced multipotency, ECM deposition, immunomodulatory capacity, and in vivo persistence, compared to both conventional 2D culture and ULA‐based spheroid culture. These properties collectively confer superior therapeutic efficacy in mouse models of inflammatory diseases. Our findings emphasize the critical influence of the culture microenvironment on stem cell phenotype and function, and position the poly‐Z matrix as a promising culture platform for stem cell–based therapies, particularly for anti‐inflammatory and regenerative medicine applications requiring high quality and immunomodulatory functions.

## 5 Experimental Section

### 5.1 Materials

2,4,6,8‐tetramethyl‐2,4,6,8‐tetravinylcyclotetrasiloxane (V4D4, 99%) was purchased from Alfa Aesar (Thermo Fisher Scientific, Ward Hill, MA). 2,4,6,8‐tetramethylcyclotetrasiloxane (TMCTS) and platinum(0)‐1,3‐divinyl‐1,1,3,3‐tetramethyldisiloxane (Karstedt's catalyst) were purchased from Sigma–Aldrich (Merck KGaA, St. Louis, MO).

### 5.2 Preparation of Poly‐Z‐Coated Cell Culture Plates

Two monomers, V4D4 (850 µL) and TMCTS (150 µL) were mixed at a molar ratio of 4:1. Karstedt's catalyst (900 µg) was then added to the mixture. The resulting solution was dispensed into each well of a 6‐well tissue culture plate (TCP) at 514 µL per well, or 5.09 mL per 100‐mm dish, and evenly spread across the bottom surface. Plates were placed in a laboratory oven at 60°C for 24 h to allow the hydrosilylation reaction to proceed. After cooling, the plates were washed sequentially with isopropyl alcohol, 70% ethanol, and distilled water to remove unreacted residues.

### 5.3 Surface Characterization

Fourier transform infrared (FT‐IR) spectra of V4D4, TMCTS, and poly‐Z were acquired using an FT‐IR spectrometer (Nicolet iS50, Thermo Fisher Scientific Instrument). Data were collected in absorbance mode with an average of 64 scans at a resolution of 0.09 cm^−1^ and recorded over the range of 400–4,000 cm^−1^.

Surface roughness was analyzed by atomic force microscopy (AFM; Dimension, Bruker) in tapping mode over a 45 × 45 µm area on TCP, ULA, and poly‐Z surfaces.

Surface wettability was analyzed using a contact angle analyzer (Surface Electro Optics, Suwon, Republic of Korea) by placing a 10 µL droplet of deionized water on the surface of TCP, ULA, and poly‐Z.

### 5.4 hADSC Culture

Human adipose‐derived stem cells (hADSCs) (StemPro, Gibco, Thermo Fisher Scientific, Waltham, MA) were cultured in low‐glucose DMEM (Gibco) supplemented with 10% fetal bovine serum (FBS; Gibco) and 1% penicillin/streptomycin (P/S; Gibco) on standard TCP according to the manufacturer's instructions. Cells were maintained at 37°C in a humidified atmosphere with 5% CO_2_. For 3D spheroid culture on poly‐Z, hADSCs were seeded at a density of 5 × 10^4^ cells/cm^2^ in low‐glucose DMEM supplemented with 10% serum replacement (SR; Gibco) and 1% P/S and cultured for 4 days. For comparison, cells were also seeded at the same density on ultra‐low attachment (ULA) plates (Corning, Corning, NY) and cultured in low‐glucose DMEM containing 10% FBS and 1% P/S for 4 days. Media were refreshed every 2 days. Cells were used within 4 passages post‐thawing to minimize passage‐related senescence. In FAK inhibition experiments, hADSCs were cultured on poly‐Z in 10% SR‐containing medium supplemented with 10 µm FAK inhibitor 14 (Sigma–Aldrich) for 4 days. The formed cell aggregates were then collected for further analysis. Cell morphology was visualized using an inverted microscope (IX53; Olympus, Tokyo, Japan).

### 5.5 Dissociation of hADSC Spheroids Into Single Cells

ULA‐cultured spheroids were dissociated using Trypsin‐EDTA (Gibco) for 10 min at 37°C. Poly‐Z‐cultured spheroids were first incubated with collagenase type IV (Gibco) for 30 min at 37°C, followed by a 10 min incubation with Trypsin‐EDTA.

### 5.6 Proliferation Measurement and Live/Dead Staining

Cell proliferation was assessed using a hemocytometer after trypan blue exclusion (Sigma–Aldrich). Spheroids were dissociated into single cells as described above prior to counting. For live/dead staining, hADSC spheroids were stained with propidium iodide (PI; Invitrogen, Thermo Fisher Scientific) and DAPI (Invitrogen) according to the manufacturer's protocol. Fluorescence images were acquired using an inverted fluorescence microscope (TI2; Nikon, Tokyo, Japan).

### 5.7 Spheroid Formation at Varying Concentrations of BSA

hADSCs were cultured on ULA plates or poly‐Z using serum‐free media supplemented with varying concentrations of BSA. Cell morphologies were observed after 24 h of culture, and spheroids were collected by centrifuging at 100 × g for 2 min. DNA was isolated from the collected spheroids using the AccuPrep Genomic DNA Extraction kit (Bioneer) according to the manufacturer's instructions. The amount of DNA was quantified using the Quant‐iT dsDNA assay kit (Invitrogen) according to the manufacturer's instructions, and the spheroid formation efficiency was calculated (DNA amounts of spheroids / DNA amounts of seeded cells). To investigate spheroid formation on albumin‐pre‐coated poly‐Z, various concentrations of BSA solutions were incubated on poly‐Z for 24 h. hADSCs were then cultured on albumin‐pre‐coated poly‐Z using serum‐free media. Cell morphology was observed after 24 h of culture, and spheroids were collected by centrifuging at 100 × g for 2 min. DNA was isolated and quantified according to the above procedure, and the spheroid formation efficiency was calculated.

### 5.8 Quantitative Real‐Time PCR (qRT‐PCR)

Total RNA was isolated from hADSCs cultured on TCP, ULA, or poly‐Z plates using Ribospin II (GeneAll Biotechnology, Seoul, Republic of Korea), following the manufacturer's instructions. qRT‐PCR was performed on a CFX96 Real‐Time PCR Detection System (Bio‐Rad, Hercules, CA) using 100 ng RNA and SB‐Green One‐Step qRT‐PCR Master Mix (LeGene Biosciences, San Diego, CA). Primer sequences are listed in Table S1.

### 5.9 Immunocytochemistry (ICC)

TCP‐cultured hADSCs were fixed in 4% paraformaldehyde (PFA; Sigma–Aldrich) for 15 min at room temperature, permeabilized with 0.2% Triton X‐100 (Sigma–Aldrich) for 15 min, washed with DPBS, and blocked with 3% BSA in DPBS for 30 min. ULA‐ and poly‐Z‐cultured spheroids were fixed in 4% PFA for 30 min, then dehydrated sequentially in 15% and 30% sucrose until they sank. Frozen blocks were embedded in FSC 22 Clear (Leica Microsystems, Wetzlar, Germany) and sectioned at 10 µm using a cryotome (CM1850; Leica). Sections were mounted on slides (Paul Marienfeld GmbH & Co. KG, Lauda‐Königshofen, Germany), permeabilized, blocked, and incubated with primary antibodies overnight at 4°C, followed by secondary antibody incubation for 1 h at room temperature. Antibody information is provided in Tables S2 and S3. DAPI staining was used to visualize nuclei. Imaging was performed using a fluorescence microscope (TI2; Nikon) and a confocal laser scanning microscope (LSM890; Carl Zeiss Microscopy GmbH, Jena, Germany).

### 5.10 Flow Cytometry

Single‐cell suspensions were prepared from TCP‐cultured cells using Trypsin‐EDTA, and from ULA‐ or poly‐Z‐cultured spheroids using the dissociation protocols described above. Cells were resuspended in FACS buffer (2% FBS in DPBS), stained with fluorophore‐conjugated antibodies for 30 min at 4°C, washed, and analyzed on a BD LSR Fortessa flow cytometer. Data were processed using FlowJo software (Tree Star Inc., Ashland, OR). The list of antibodies used is provided in Table S2.

### 5.11 Differentiation of hADSCs

For all differentiation protocols, ULA‐ and poly‐Z‐cultured spheroids were dissociated prior to induction.

#### 5.11.1 Adipogenic Differentiation

Cells were cultured for 14 days in DMEM/F‐12 with 10% FBS, 1% P/S, 500 µm IBMX, 1 µm dexamethasone, 10 µm insulin, and 200 µm indomethacin [32]. *PPAR‐γ* gene expression was measured, and Oil Red O staining was performed.

#### 5.11.2 Osteogenic Differentiation

Cells were cultured for 14 days in high‐glucose DMEM with 10% FBS, 1% P/S, 10 nm dexamethasone, 50 µm 2‐phospho‐ascorbic acid, and 10 mm β‐glycerophosphate [32]. *RUNX2* gene expression was measured, and Alizarin Red S staining was performed.

#### 5.11.3 Neurogenic Differentiation

Cells were cultured in high‐glucose DMEM with 1% FBS, 1% P/S, and 100 ng/mL bFGF for 7 days, followed by 10 µm forskolin for 7 days [63]. *TUBB3* and *NES* gene expression and β‐III‐tubulin protein expression were assessed by qRT‐PCR and ICC.

#### 5.11.4 Hepatogenic Differentiation

Cells were cultured in high‐glucose DMEM with 20 ng/mL EGF and 10 ng/mL bFGF for 48 h, followed by medium supplemented with 4.9 mm nicotinamide for 4 weeks [64]. *ALB* gene expression and Albumin protein expression were analyzed by qRT‐PCR and ICC.

### 5.12 Animals

Seven‐week‐old C57BL/6 mice (Samtaco Bio Korea, Seoul, Republic of Korea) were housed under specific pathogen‐free conditions at KAIST. All procedures were approved by the KAIST Institutional Animal Care and Use Committee (KA2024‐167‐v1). Mice were acclimated for 1 week prior to experiments. All surgeries were performed under isoflurane anesthesia.

### 5.13 DSS‐Induced Acute Colitis Model

Mice were randomly assigned to normal or colitis groups. Colitis was induced with 3% DSS (MP Biomedicals, Irvine, CA) in drinking water for 5 days, followed by 3 days of regular water. On day 2, 1 × 10^6^ hADSCs (TCP and ULA or poly‐Z‐cultured spheroids) premixed with Vitrogel (TheWell Bioscience, North Brunswick, NJ) were injected intraperitoneally. Disease activity index (DAI; Table S4) was assessed daily. On day 9, colons were collected, measured, and processed for histology or cytokine analysis. For cytokine quantification, colon tissue was homogenized in T‐PER buffer with protease inhibitors (Thermo Fisher Scientific), centrifuged, and analyzed for protein concentration (Micro BCA Assay; Thermo Fisher Scientific). Fixed colon samples were embedded in paraffin and stained with H&E. Histological scoring was performed based on standard criteria (Table S5) [65].

### 5.14 Acetaminophen‐Induced Acute Liver Injury Model

After 16 h fasting, mice were intraperitoneally injected with 300 mg/kg acetaminophen (APAP; Sigma). Two hours later, 1 × 10^6^ hADSCs (TCP and ULA or poly‐Z‐cultured spheroids) premixed with Vitrogel were injected intraperitoneally. Serum was collected daily for 4 days, and livers were harvested for histology. Serum ALT and AST levels were quantified at the Korean Pathology Technical Center (KP&T, Seoul, Republic of Korea). Liver sections were fixed, embedded, and stained with H&E. Necrotic areas were quantified by randomly selecting 3 areas per H&E slide using Image J.

### 5.15 Enzyme‐Linked Immunosorbent Assay (ELISA)

For in vitro cytokine analysis, hADSCs were cultured on TCP, ULA, or poly‐Z for 4 days, then incubated for 24 h in serum‐free medium. Supernatants were analyzed using DuoSet ELISA kits (R&D Systems, Inc., Minneapolis, MN) to quantify hIL‐10 (DY217B), hHGF (DY294), hIDO (DY6030B), and PGE2 (KGE004B). Cytokine levels in colon tissue homogenates were analyzed for IL‐6 (DY406), IL‐1β (DY401), TNF‐α (DY410), IFN‐γ (DY258), IL‐10 (DY417), and TGF‐β (DY1679) using corresponding Mouse DuoSet ELISA kits. All procedures followed the manufacturer's instructions.

### 5.16 In Vivo Cell Tracking

hADSCs were labeled with 5 µm DiR dye (Biotium, Fremont, CA) per manufacturer's protocol. Mice were shaved one day before injection. On day 0, 1 × 10^6^ DiR‐labeled hADSCs (TCP or spheroids) mixed with Vitrogel were injected subcutaneously. Fluorescence near injection sites was monitored using an IVIS imaging system (Perkin Elmer, Waltham, MA) on days 1, 3, 5, and 7.

### 5.17 Statistical Analysis

Data are presented as mean ± standard deviation (SD). Statistical comparisons were made using Student's t‐test (two groups) or one‐way ANOVA with Tukey's post hoc test (three or more groups), using GraphPad Prism v8.2. A p‐value < 0.05 was considered statistically significant.

## Author Contributions

C.S. took the lead on the project, handling conceptualization, data curation, formal analysis, and methodology. They were also responsible for the investigation, visualization, and the complete drafting and editing of the manuscript. D.K. contributed to the conceptualization, methodology, and investigation, while J.S. assisted with the methodology, investigation, and visualization. S.K., Y.S., and A.T.R. provided support with the methodology. Finally, S.J. oversaw project administration and supervision, while also contributing to the conceptualization and the drafting and editing of the manuscript.

## Funding

This work was supported by the Korean Fund for Regenerative Medicine (KFRM) grant funded by the Korea government (the Ministry of Science and ICT, the Ministry of Health and Welfare) (25A0107L1), by the InnoCORE program of the Ministry of Science and ICT (N10250153), and by the National Research Foundation of Korea (NRF) grant funded by the Korea government (MSIT) (RS‐2018‐NR030951).

## Conflicts of Interest

The authors declare no conflicts of interest.

## Supporting information




**Supporting File**: advs75051‐sup‐0001‐SuppMat.docx.

## Data Availability

The data that support the findings of this study are available from the corresponding author upon reasonable request.
