# Supplementary material for: Polymer Matrix‐Based 3D Culture Significantly Enhances the Differentiation and Immunomodulatory Functions of Human Adipose‐Derived Stem Cells
Source: Adv Sci (Weinh). 2026 Mar 31;13(34):e18704. doi: 10.1002/advs.202518704 (PMC13285109; doi:10.1002/advs.202518704)
Supplement: Supplementary file 1 — Supporting File: advs75051‐sup‐0001‐SuppMat.docx. [file ADVS-13-e18704-s001.docx]

**Supporting Information**

**Polymer Matrix-Based 3D Culture Significantly Enhances the Differentiation and Immunomodulatory Functions of Human Adipose-Derived Stem Cells**

Changjin Seo^1,2,3^, Dohyeon Kim^1,2^, Junhyuk Song^1,2^, Sunyoung Kim^1,2^, Youngju Son^1,2^, Afia Tasnim Rahman^1,2^, Sangyong Jon^1,2,*^

^1^Department of Biological Sciences, KAIST Institute for the BioCentury, Korea Advanced Institute of Science and Technology (KAIST), 291 Daehak-ro, Daejeon 34141, Republic of Korea

^2^Center for Precision Bio-Nanomedicine, Korea Advanced Institute of Science and Technology (KAIST), 291 Daehak-ro, Daejeon 34141, Republic of Korea

^3^InnoCORE AI-CRED Institute, Korea Advanced Institute of Science and Technology (KAIST), 291 Daehak-ro, Daejeon, 34141, Republic of Korea

*Address correspondence to: syjon@kaist.ac.kr (Sangyong Jon)

Supplementary figures: S1-S11

Supplementary tables: S1-S5


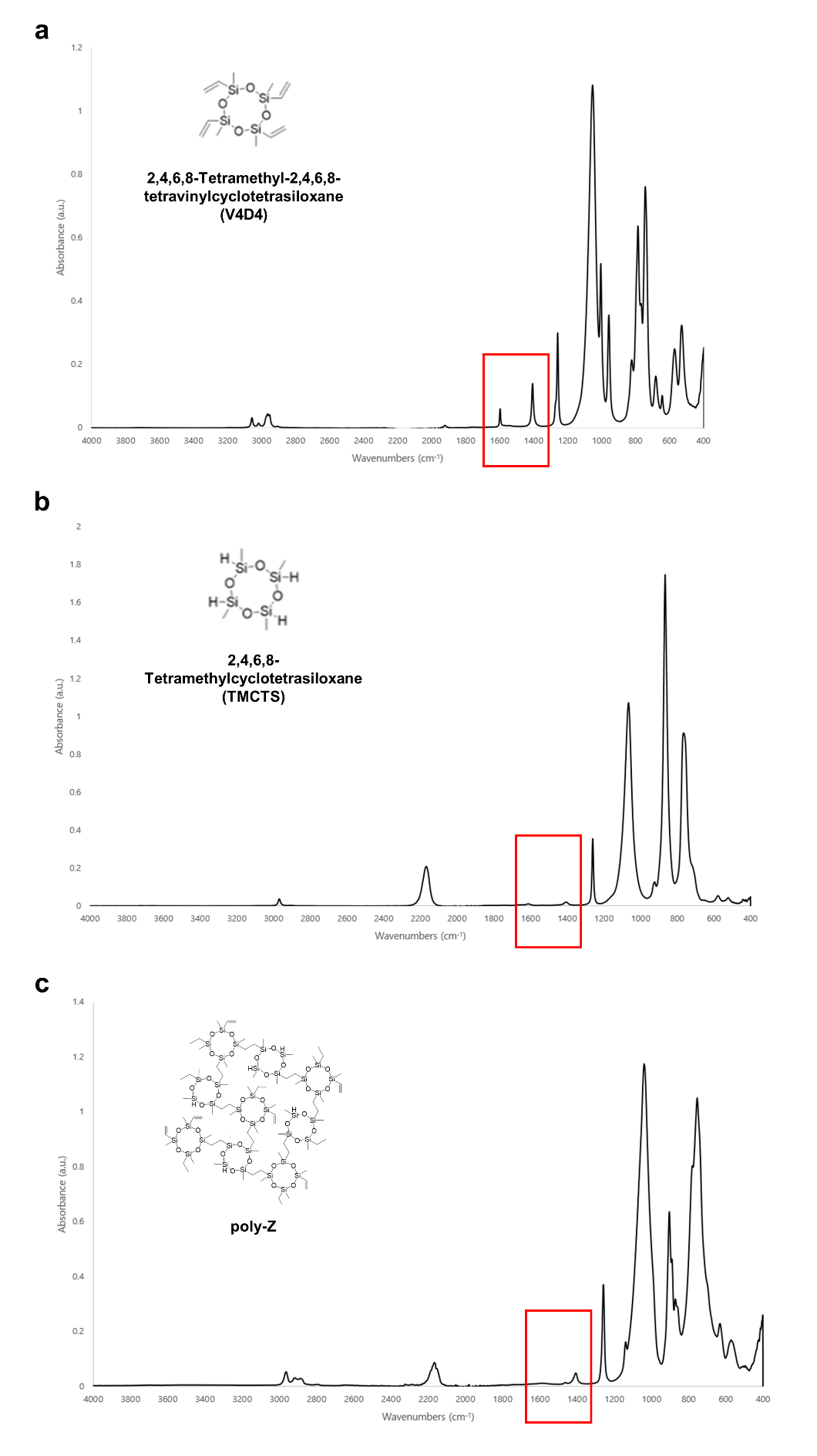


**Figure S1. FT-IR spectra of V4D4 monomer, TMCTS monomer, and poly-Z.** The red boxes indicate 1400 cm^-1^ and 1600 cm^-1^, which are representative peaks of the vinyl group.


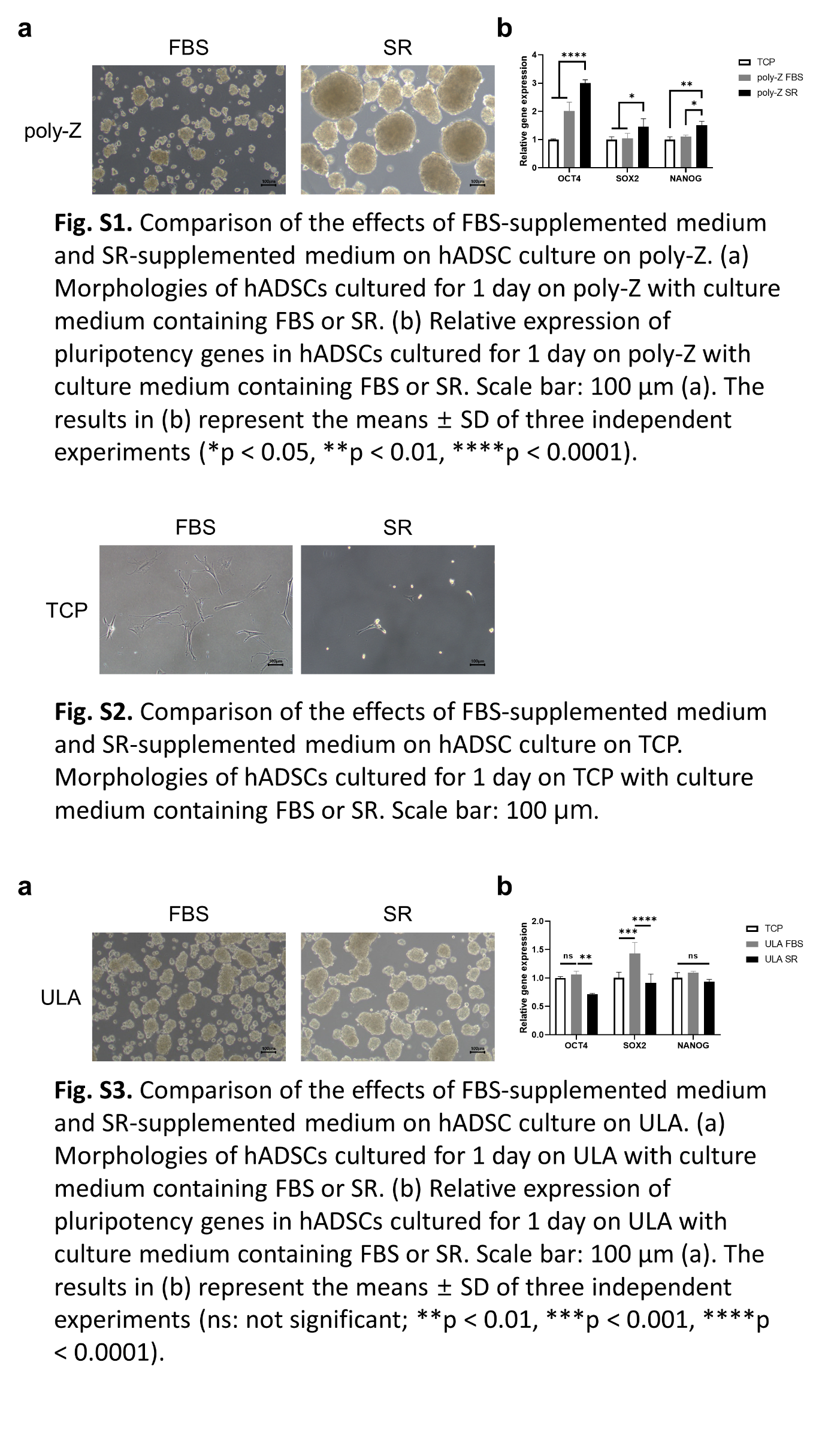


**Figure S2. Effects of FBS- and SR-supplemented media on hADSC culture on poly-Z.** (a) Representative morphologies of hADSCs cultured for 1 day on poly-Z in media supplemented with either fetal bovine serum (FBS) or serum replacement (SR). (b) Relative expression levels of pluripotency-related genes in hADSCs cultured for 1 day on poly-Z with FBS- or SR-supplemented medium. Scale bar: 100 µm (a). Data in (b) is presented as mean ± SD of three independent experiments (n = 3). (*p < 0.05; **p < 0.01; ****p < 0.0001).


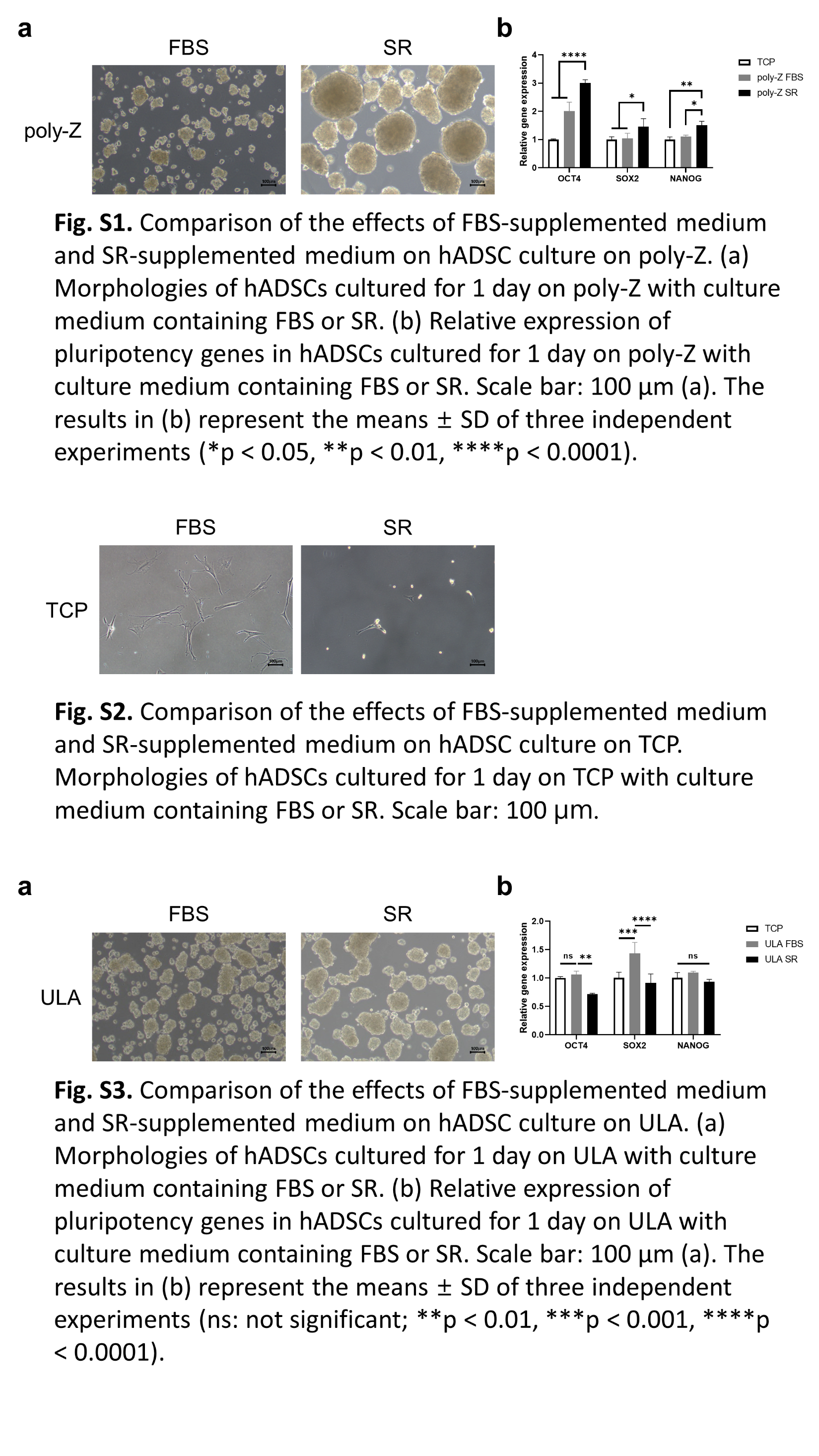


**Figure S3.** Effects of FBS- and SR-supplemented media on hADSC culture on TCP. Representative morphologies of hADSCs cultured for 1 day on tissue culture plates (TCP) in media supplemented with either fetal bovine serum (FBS) or serum replacement (SR). Scale bar: 100 µm.


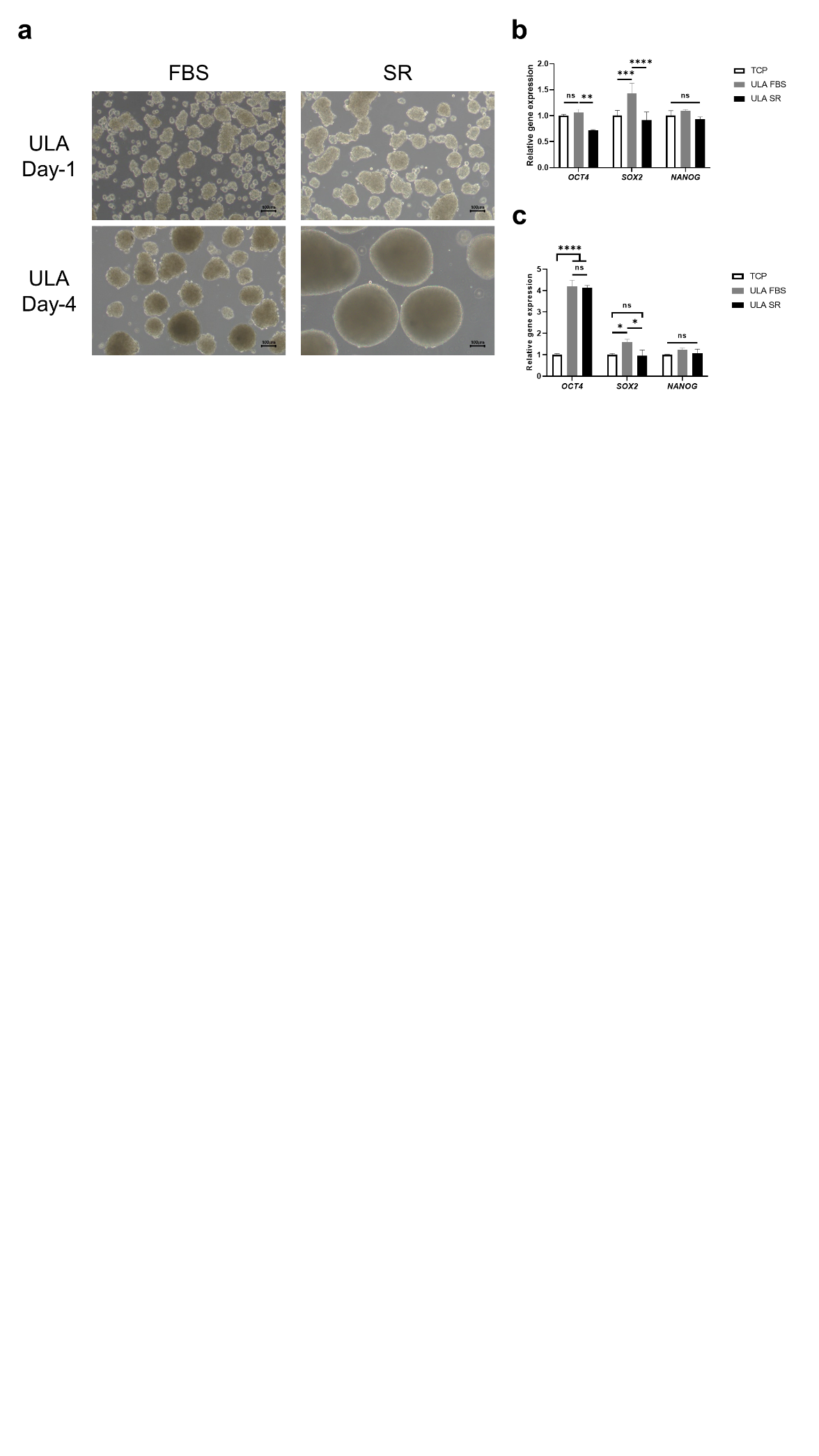


**Figure S4. Effects of FBS- and SR-supplemented media on hADSC culture on ULA plates.** (a) Representative morphologies of hADSCs cultured for 1 and 4 days on ultra-low attachment (ULA) plates in media supplemented with either fetal bovine serum (FBS) or serum replacement (SR). (b) Relative expression levels of pluripotency-related genes in hADSCs cultured for 1 day on ULA plates with FBS- or SR-supplemented medium. (c) Relative expression levels of pluripotency-related genes in hADSCs cultured for 4 days on ULA plates with FBS- or SR-supplemented medium. Scale bar: 100 µm (a). Data in (b, c) are presented as mean ± SD of three independent experiments (n = 3). (ns: not significant; *p < 0.05; **p < 0.01; ***p < 0.001; ****p < 0.0001).


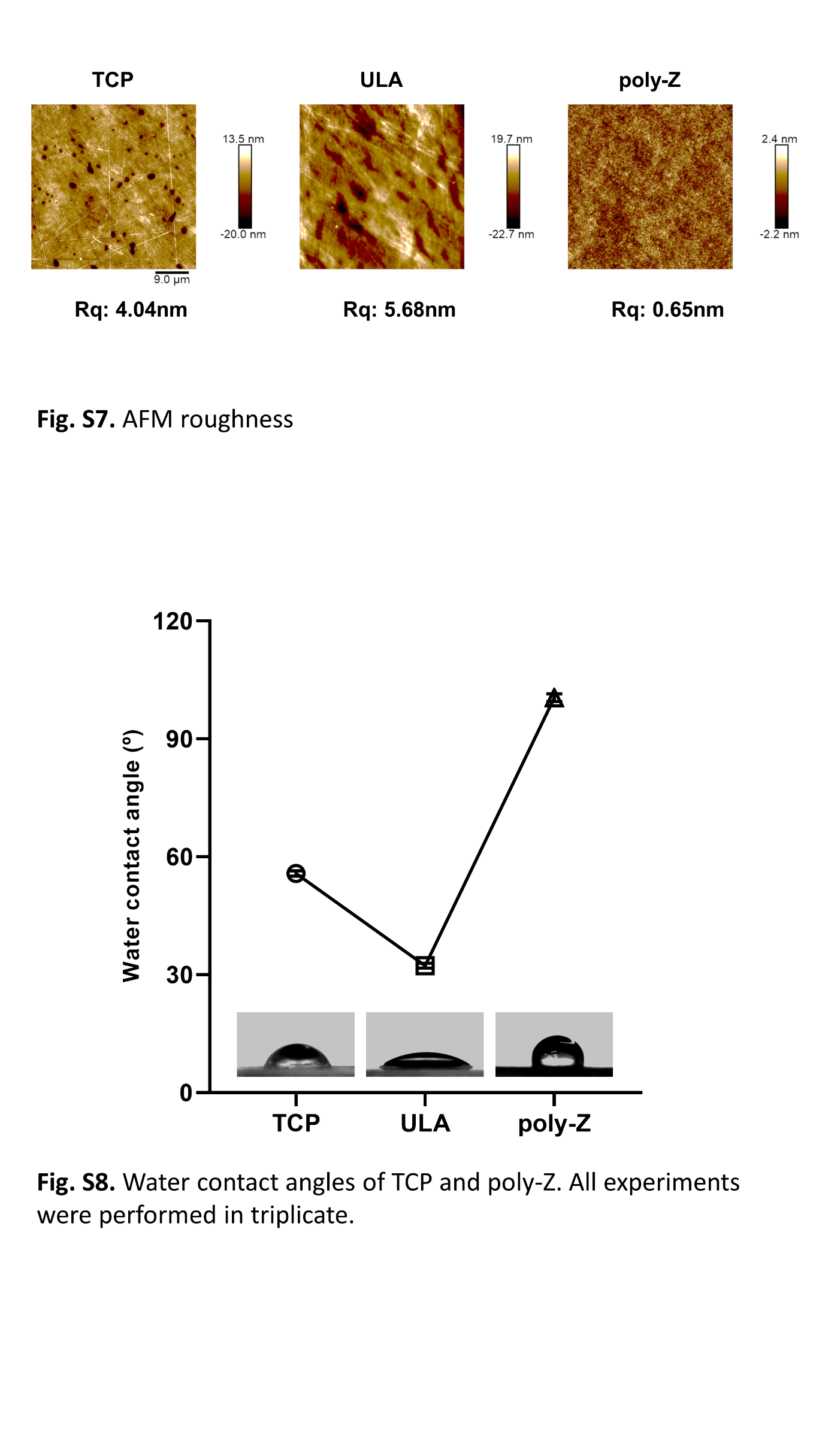


**Figure S5. Surface roughness of TCP, ULA, and poly-Z plate.**


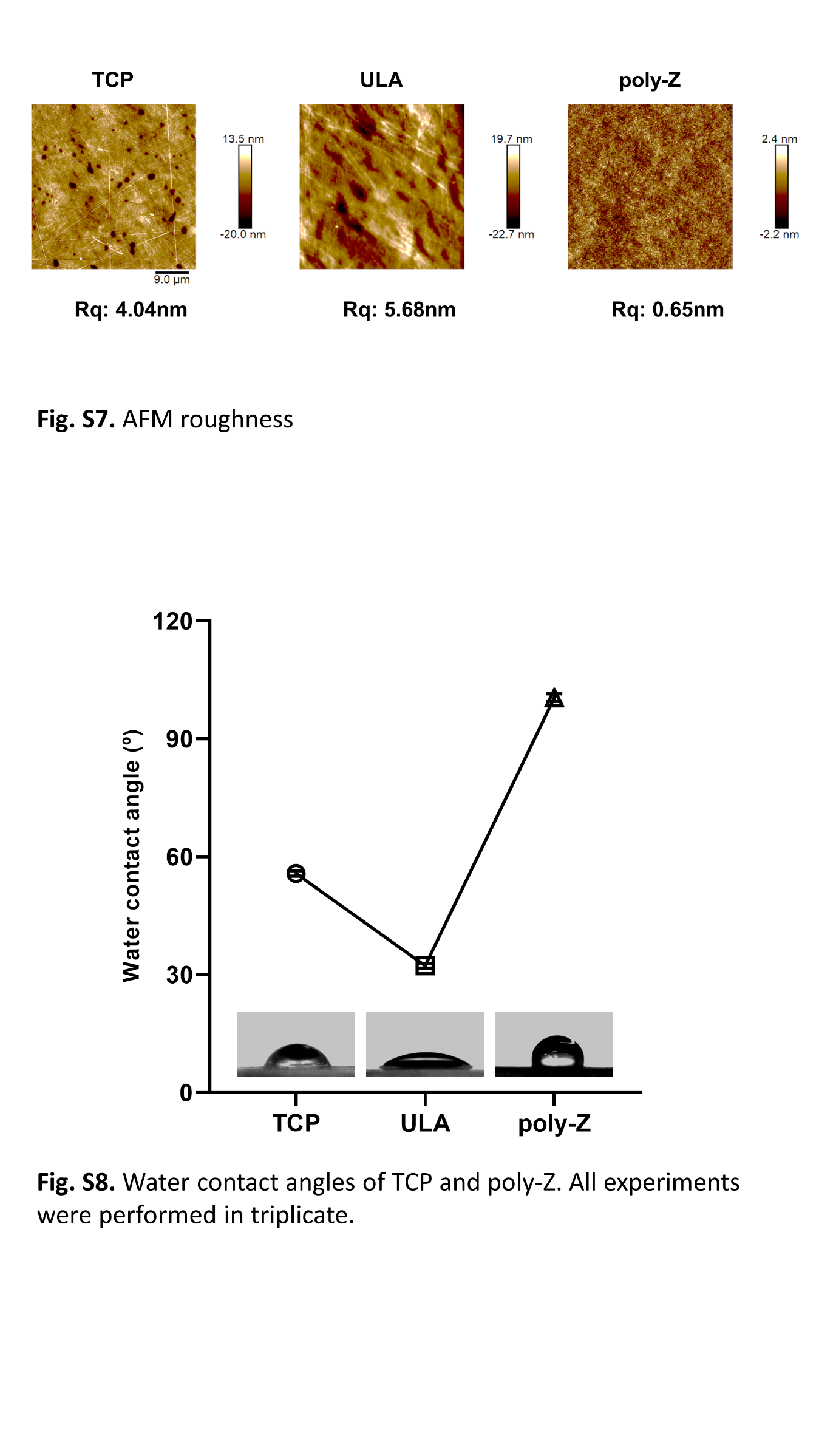


**Figure S6. Water contact angles of TCP, ULA, and poly-Z. All experiments were performed in triplicate (n = 3).**


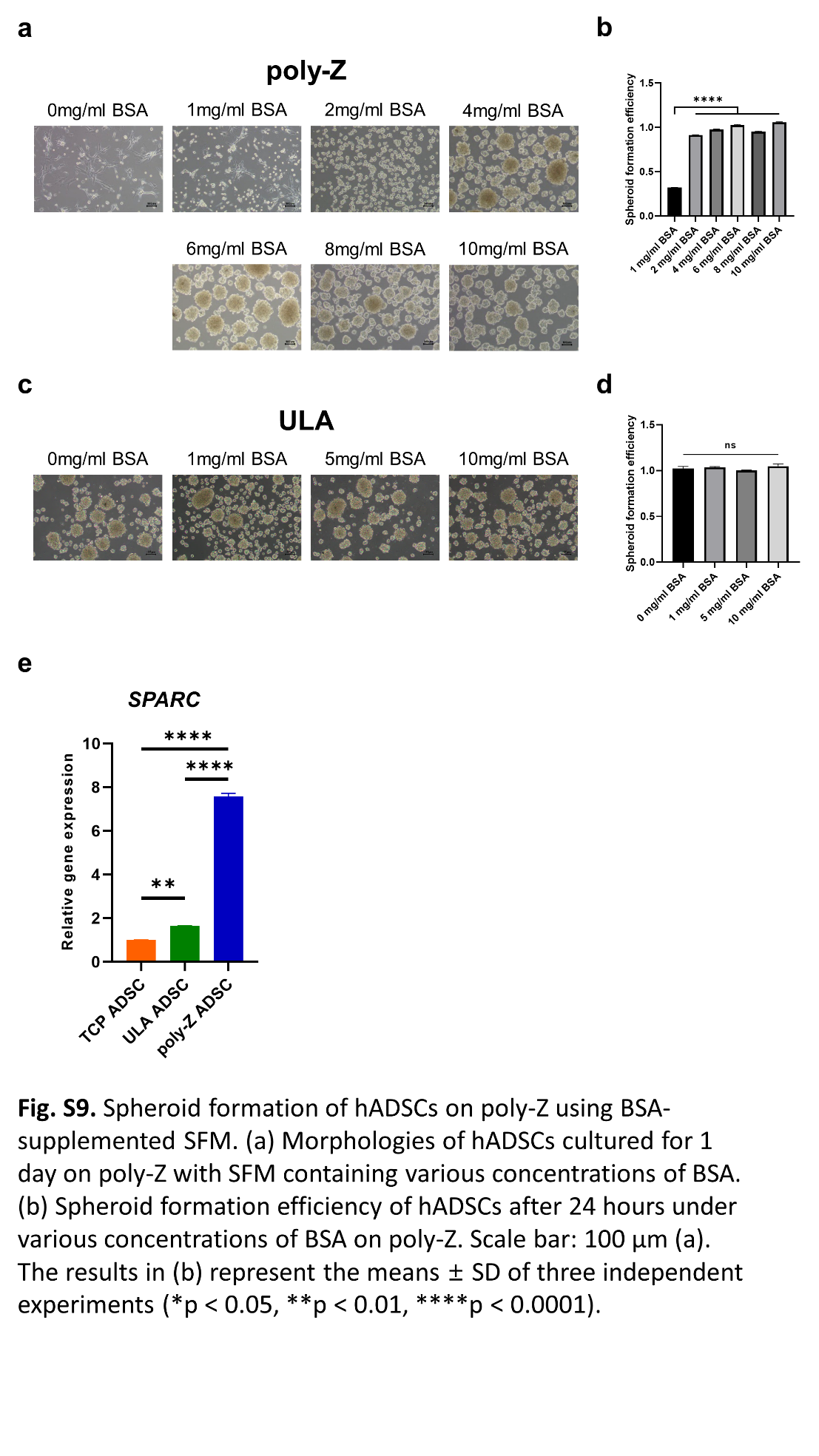


**Figure S7. Spheroid formation of hADSCs on poly-Z and ULA plate using BSA-supplemented serum-free media. (a)** Morphologies of hADSCs cultured for 24 hours on poly-Z with serum-free media containing various concentrations of BSA. (b) Spheroid formation efficiency of hADSCs after 24 hours under various concentrations of BSA on poly-Z. (c) Morphologies of hADSCs cultured for 24 hours on ULA plates with serum-free media containing various concentrations of BSA. (d) Spheroid formation efficiency of hADSCs after 24 hours under various concentrations of BSA on ULA plates. (e) Relative mRNA expression of *SPARC* gene in hADSCs cultured for 4 days on TCP, ULA, and poly-Z. Scale bar: 100 µm (a, c). Data in (b, d, e) are presented as mean ± SD of three independent experiments (n = 3). (ns: not significant; **p < 0.01; ****p < 0.0001).


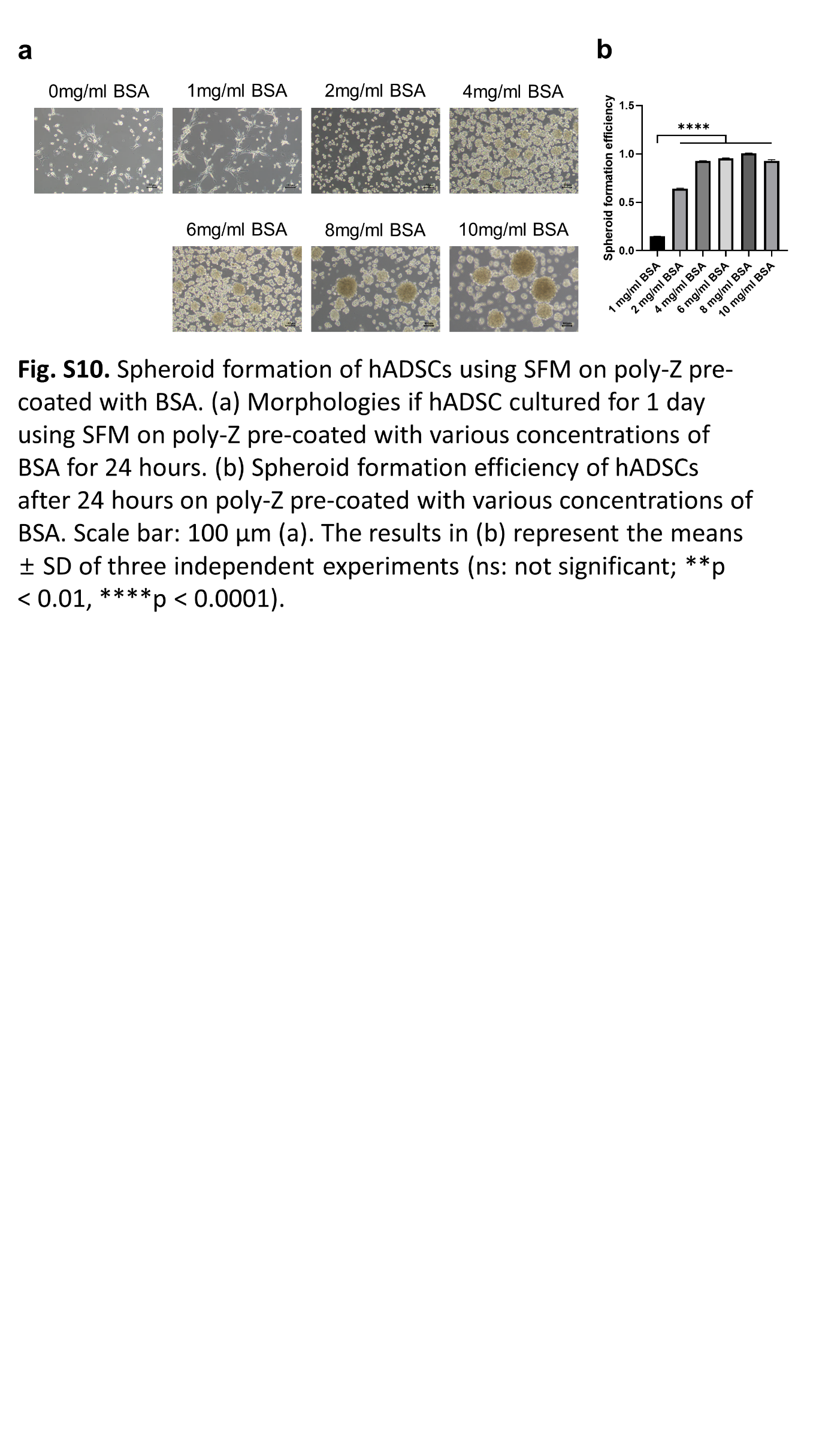


**Figure S8.** Spheroid formation of hADSCs using serum-free media on poly-Z pre-coated with BSA. (a) Morphologies of hADSCs cultured for 24 hours using serum-free media on poly-Z pre-coated with various concentrations of BSA for 24 hours. (b) Spheroid formation efficiency of hADSCs after 24 hours under serum-free media on poly-Z pre-coated with various concentrations of BSA. Scale bar: 100 µm (a). The results in (b) represent the means ± SD of three independent experiments (n = 3). (****p < 0.0001).


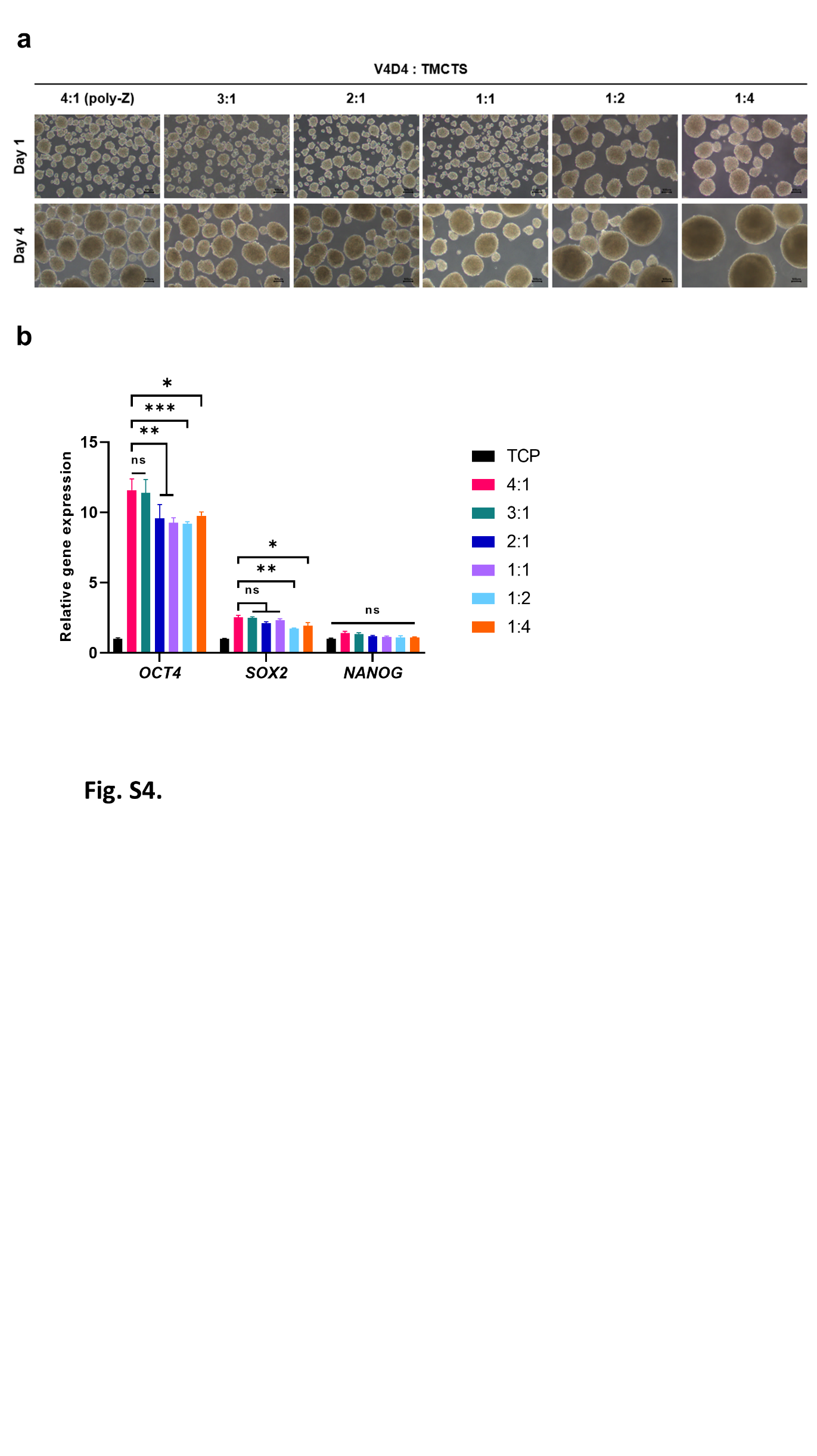


**Figure S9. hADSC spheroids generated in polymer matrices synthesized with various ratios of V4D4 and TMCTS.** (a) Representative morphologies of hADSCs cultured for 1 and 4 days on polymer matrices synthesized with various ratios of V4D4 and TMCTS. (b) Relative expression levels of pluripotency-related genes in hADSCs cultured for 4 days on polymer matrices synthesized with various ratios of V4D4 and TMCTS. Scale bar: 100 µm (a). Data in (b) is presented as mean ± SD of three independent experiments (n = 3). (ns: not significant; *p < 0.05; **p < 0.01; ***p < 0.001).


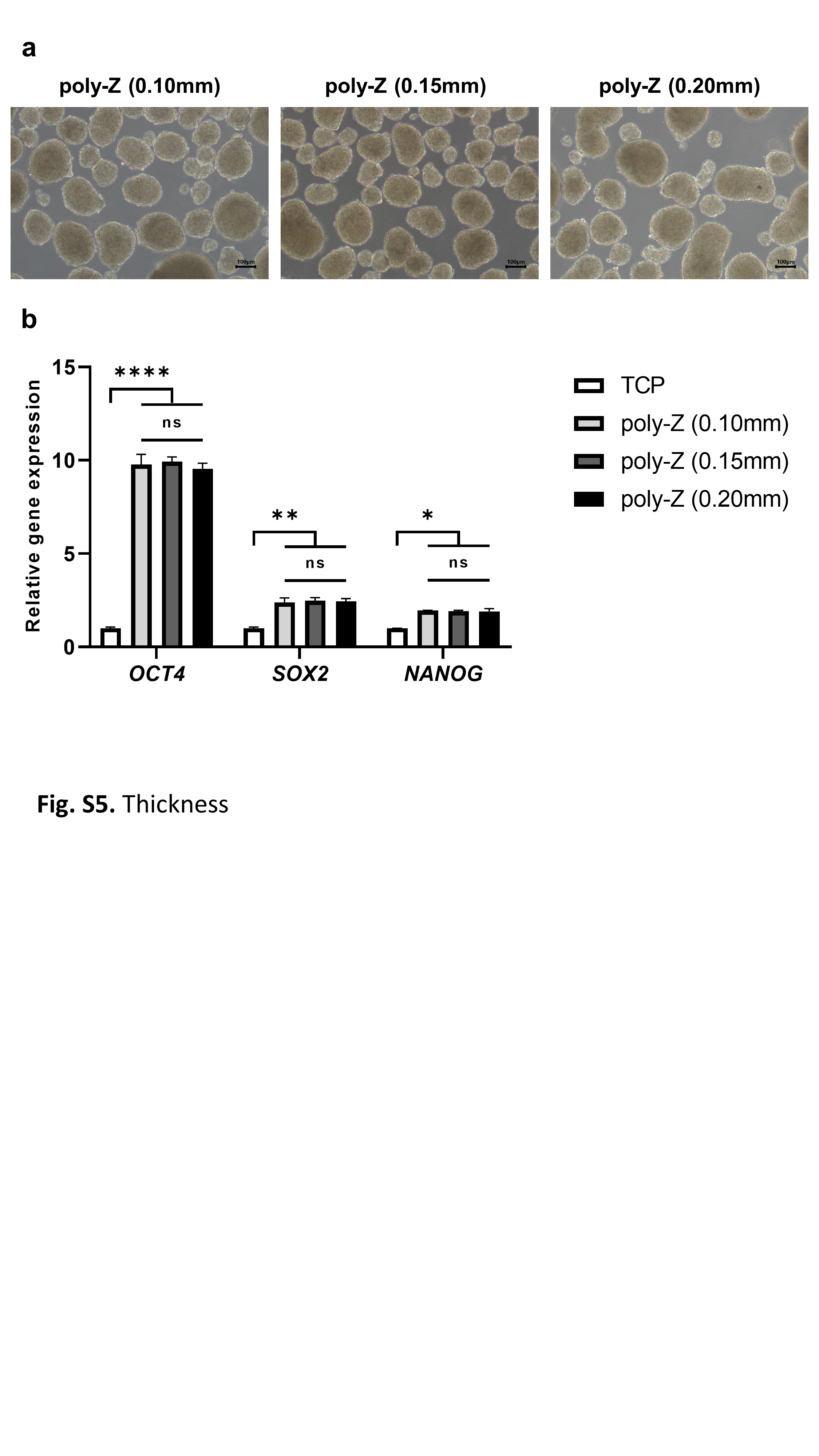


**Figure S10. Effect of poly-Z thickness on hADSC culture. (a) Representative morphologies of hADSCs cultured for 4 days on poly-Z with thicknesses of 0.1, 0.15, and 0.2 mm. (b)** Relative expression levels of pluripotency-related genes in hADSCs cultured for 4 days on poly-Z with thicknesses of 0.1, 0.15, and 0.2 mm. Scale bar: 100 µm (a). Data in (b) is presented as mean ± SD of three independent experiments (n = 3). (ns: not significant; *p < 0.05; **p < 0.01; ****p < 0.0001).


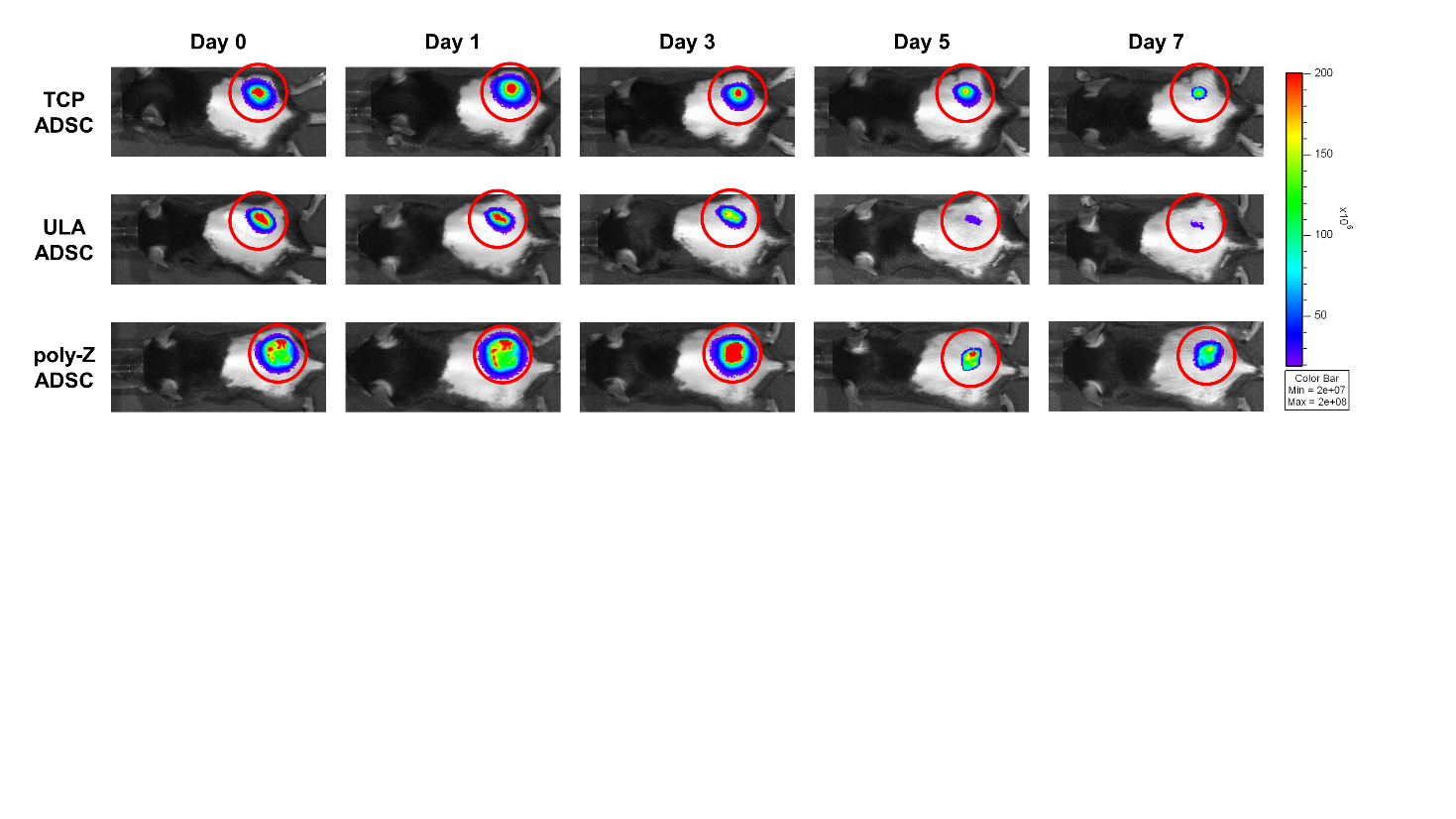


**Figure S11. In vivo persistence of transplanted hADSCs and hADSC spheroids.** In vivo imaging system (IVIS) images showing the retention of DiR-labeled hADSCs and hADSC spheroids at the subcutaneous injection site on days 1, 3, 5, and 7 post-injection.

**Table S1. Sequences of primers used for qRT-PCR**

| **Human gene** | **Primer pair** | **Primer sequences (5’ – 3’)** |
| --- | --- | --- |
| *GAPDH* | Forward primer | TGGACCTGACCTGCCGTCTA |
|  | Reverse primer | CCCTGTTGCTGTAGCCAAATTC |
| *OCT4* | Forward primer | AAGCGAACCAGTATCGAGAACC |
|  | Reverse primer | CTGATCTGCTGCAGTGTGGGT |
| *SOX2* | Forward primer | GGCAATAGCATGGCGAGC |
|  | Reverse primer | TTCATGTGCGCGTAACTGTC |
| *NANOG* | Forward primer | AATACCTCAGCCTCCAGCAGATG |
|  | Reverse primer | TGCGTCACACCATTGCTATTCTTC |
| *FN1* | Forward primer | GCCACCCCCATAAGGCATAG |
|  | Reverse primer | CCGTGTGGGTACAGGTGATAG |
| *COL1A1* | Forward primer | AAAACCACCAAGACCTCCCG |
|  | Reverse primer | TTACAGGAAGCAGACAGGGC |
| *LAMA1* | Forward primer | ATATGAGCCCAAAACCGCCA |
|  | Reverse primer | GCGTTCCCGTCAACAATCAG |
| *LAMB1* | Forward primer | GCTTTCAGTTTCTTAGCCCTGTG |
|  | Reverse primer | ATGAGAAGGTCGCCCGTG |
| *LAMC1* | Forward primer | ACCTACTCCAAGGCAAACCG |
|  | Reverse primer | CCCCAGTGAGGGGAGAAATG |
| *PPAR-γ* | Forward primer | GAGGGCGATCTTGACAGGAAA |
|  | Reverse primer | GGGGTGATGTGTTTGAACTTGA |
| *RUNX2* | Forward primer | GTCCCCGTCCATCCACTCTA |
|  | Reverse primer | TCTGAAGCACCTGAAATGCG |
| *TUBB3* | Forward primer | GAGCAACATGAACGACCTGG |
|  | Reverse primer | TCGTCGTCTTCGTACATCTCG |
| *NES* | Forward primer | CACTGGAAGTGATGCCCCTT |
|  | Reverse primer | CAGGATCGGGGTGTACGTTG |
| *ALB* | Forward primer | TTTATGCCCCGGAACTCCTT |
|  | Reverse primer | ACAGGCAGGCAGCTTTATCA |
| *IL-10* | Forward primer | GGGCACCCAGTCTGAGAAC |
|  | Reverse primer | TCACTCTGCTGAAGGCATCTC |
| *IDO* | Forward primer | CAACCCCCAGCTATCAGACG |
|  | Reverse primer | AAAGCACTGAAAGACGCTGC |
| *NOS2* | Forward primer | CCTCCCATCCTTGCATCCTC |
|  | Reverse primer | CAAACACCAAGGTCATGCGG |
| *TGF-β* | Forward primer | GGAAATTGAGGGCTTTCGCC |
|  | Reverse primer | CCGGTAGTGAACCCGTTGAT |
| *COX-2* | Forward primer | GCCAAGCACTTTTGGTGGAG |
|  | Reverse primer | GGGACAGCCCTTCACGTTAT |
| *HGF* | Forward primer | GAATTCCATGTCAGCGTTGGG |
|  | Reverse primer | AACACCAGGGTGATTCAGACC |
| *ITGA1* | Forward primer | CCGATCCAGAAAATGGGCCT |
|  | Reverse primer | CTGGGATCGGACAATCAGCA |
| *ITGA5* | Forward primer | TCTCAGTGGAGTTTTACCGGC |
|  | Reverse primer | ACCCCAAGGACAGAGGTAGA |
| *ITGAV* | Forward primer | CGCTTCTTCTCTCGGGACTC |
|  | Reverse primer | GAAGAAATCCACGGCGAAGC |
| *ITGB1* | Forward primer | ACCGTAGCAAAGGAACAGCA |
|  | Reverse primer | AAATGTCTGTGGCTCCCCTG |
| *ITGB3* | Forward primer | GCTTGCCCATGTTTGGCTAC |
|  | Reverse primer | CCGTGACACACTCTGCTTCT |
| *ITGB5* | Forward primer | AGTTTCAGAGCGAGCGATCC |
|  | Reverse primer | TTGTTGAAGGTGAAGTCCACAG |
| *SPARC* | Forward primer | GATGGTGCAGAGGAAACCGA |
|  | Reverse primer | CGTGTTTGCAGTGGTGGTTC |

**Table S2. List of primary antibodies**

| **Antibodies** | **Manufacturers**  **(Catalogue No.)** | **Applications** | **Dilution** |
| --- | --- | --- | --- |
| Anti-CD19, PE | BioLegend  (302207) | FC | 1/20 |
| Anti-CD29, PE | BioLegend  (303003) | FC | 1/20 |
| Anti-CD44, APC | Invitrogen  (17-0441-82) | FC | 1/300 |
| Anti-CD45, APC | BioLegend  (304011) | FC | 1/20 |
| Anti-CD90, FITC | BioLegend  (328107) | FC | 1/20 |
| Anti-CD105, FITC | BioLegend  (323203) | FC | 1/20 |
| Anti-Collagen Type 1 (host: goat) | Sigma Aldrich  (AB758) | ICC | 1/20 |
| Anti-Laminin (host: rabbit) | Abcam  (ab11575) | ICC | 1/100 |
| Anti-Fibronectin (host: rabbit) | Abcam  (ab2413) | ICC | 1/100 |
| Anti-OCT4 (host: rabbit) | Invitrogen  (A24867) | ICC | 1/200 |
| Anti-SOX2 (host: rat) | Invitrogen  (A24759) | ICC | 1/100 |
| Anti-β-III-tubulin (host: rabbit) | Abcam  (ab18207) | ICC | 1/200 |
| Anti-Serum Albumin (host: mouse IgG) | R&D SYSTEMS  (MAB1455) | ICC | 1/200 |
| FC, flow cytometry; ICC, immunocytochemistry | | | |

**Table S3. List of secondary antibodies**

| **Antibodies** | **Manufacturers**  **(Catalogue No.)** | **Applications** | **Dilution** |
| --- | --- | --- | --- |
| Alexa Fluor 488 donkey anti-goat | Invitrogen  (A11055) | ICC | 1/250 |
| Alexa Fluor 488 goat anti-rabbit | Abcam  (ab150081) | ICC | 1/500 |
| Alexa Fluor 594 donkey anti-rabbit | Invitrogen  (A24870) | ICC | 1/250 |
| Alexa Fluor 488 donkey anti-rat | Invitrogen  (A24876) | ICC | 1/250 |
| Alexa Fluor 594 goat anti-mouse IgG | Abcam  (ab150116) | ICC | 1/500 |
| ICC, immunocytochemistry | | | |

**Table S4. Criteria for disease activity index (DAI) scoring**

| **Score** | **Body weight loss (%)** | **Stool consistency** | **Occult/gross rectal bleeding** |
| --- | --- | --- | --- |
| 0 | None | Normal | Negative |
| 1 | 1-5 | Loose stools | Negative |
| 2 | 5-10 | Loose stools | Hemoccult positive |
| 3 | 10-20 | Diarrhea | Hemoccult positive |
| 4 | >20 | Diarrhea | Gross bleeding |

**Table S5. Criteria for colon histological damage scoring**

| **Features** | | **Score** | **Description** |
| --- | --- | --- | --- |
| **Mucosal Epithelium** | **Epithelial surface** | 0 | No mucosal inflammation  Prolonged epithelial cells |
|  |  | 1 | Destruction of barrier  < 10% loss of epithelial surface |
|  |  | 2 | Ulcer  10~30% loss of epithelial surface |
|  |  | 3 | Ulcer  30~60% loss of epithelial surface |
|  |  | 4 | Ulcer  > 60% loss of epithelial surface |
|  | **Crypt** | 0 | No mucosal inflammation  Intact crypts |
|  |  | 1 | Destruction of barrier  < 10% loss of crypts |
|  |  | 2 | Ulcer  10~20% loss of crypts |
|  |  | 3 | Ulcer  > 20% loss of crypts |
| **Cell infiltration and edema** | | 0 | None |
|  |  | 1 | Mild infiltration |
|  |  | 2 | Moderate infiltration |
|  |  | 3 | Severe infiltration |
| **Depletion of goblet cells** | | 1 | Absent |
|  |  | 0 | Present |
